# Genetic diversity and selection signatures in maize landraces compared across 50 years of in situ and ex situ conservation

**DOI:** 10.1038/s41437-021-00423-y

**Published:** 2021-03-30

**Authors:** Francis Denisse McLean-Rodríguez, Denise Elston Costich, Tania Carolina Camacho-Villa, Mario Enrico Pè, Matteo Dell’Acqua

**Affiliations:** 1 Institute of Life Sciences, Scuola Superiore Sant’Anna, Pisa, Italy; 2 Germplasm Bank, Genetic Resources Program, International Maize and Wheat Improvement Center (CIMMYT), Texcoco, Estado de México Mexico; 3 Socioeconomic Program, International Maize and Wheat Improvement Center (CIMMYT), Texcoco, Estado de México Mexico; 4 Present Address: Lincoln Institute for Agri-Food Technology, University of Lincoln, Lincoln, UK

**Keywords:** Agricultural genetics, Population genetics

## Abstract

Genomics-based, longitudinal comparisons between ex situ and in situ agrobiodiversity conservation strategies can contribute to a better understanding of their underlying effects. However, landrace designations, ambiguous common names, and gaps in sampling information complicate the identification of matching ex situ and in situ seed lots. Here we report a 50-year longitudinal comparison of the genetic diversity of a set of 13 accessions from the state of Morelos, Mexico, conserved ex situ since 1967 and retrieved in situ from the same donor families in 2017. We interviewed farmer families who donated in situ landraces to understand their germplasm selection criteria. Samples were genotyped by sequencing, producing 74,739 SNPs. Comparing the two sample groups, we show that ex situ and in situ genome-wide diversity was similar. In situ samples had 3.1% fewer SNPs and lower pairwise genetic distances (*F*_st_ 0.008–0.113) than ex situ samples (*F*_st_ 0.031–0.128), but displayed the same heterozygosity. Despite genome-wide similarities across samples, we could identify several loci under selection when comparing in situ and ex situ seed lots, suggesting ongoing evolution in farmer fields. Eight loci in chromosomes 3, 5, 6, and 10 showed evidence of selection in situ that could be related with farmers’ selection criteria surveyed with focus groups and interviews at the sampling site in 2017, including wider kernels and larger ear size. Our results have implications for ex situ collection resampling strategies and the in situ conservation of threatened landraces.

## Introduction

Agrobiodiversity conservation is a key endeavor to support the sustainability of traditional and modern agricultural systems. Both farmers and breeders harness biodiversity by identifying traditional and improved crop varieties adapted to heterogeneous environments (Mancini et al. 2017; Brauner et al. 2019) and to evolving biotic and abiotic stresses (Akem et al. 2000; Dwivedi et al. 2016). Diverse culinary and cultural uses have been developed with this diversity (Fernández-Suárez et al. 2013), and farmers can rely on it, especially in traditional settings, to retain their autonomy for seed production (Hoogendoorn et al. 2018). However, the conservation of agrobiodiversity in the agricultural landscape is threatened by the expansion of modern agricultural production systems (including improved varieties), urbanization, climate change, environmental degradation, changes in consumers’ preferences, natural disasters and social conflicts (van de Wouw et al. 2009; McLean-Rodríguez et al. 2019).

Ex situ (i.e., in genebanks) and in situ (i.e., in farmers’ fields) conservation represent complementary efforts to safeguard agrobiodiversity. Genebanks established worldwide store representative germplasm samples of crop species, their wild relatives and wild plant species. Genebanks provide researchers access to this germplasm for evaluation and integration into modern breeding programs and for conservation purposes (Noriega et al. 2019). Simultaneously, they grant farmers access to germplasm in case of unexpected losses (Westengen et al. 2018). In situ conservation complements genebanks in maintaining local intra- and inter-species agrobiodiversity. By cultivating and propagating crops in their fields, farmers facilitate the emergence of novel variation, and realize crop adaptation and evolution based on environmental and cultural drivers (Bellon et al. 2018).

Under this framework, comparing ex situ and in situ conservation strategies can contribute to a better understanding of their underlying effects on the maintenance of agrobiodiversity and to increase their effectiveness. Previous comparisons between these two conservation strategies in different crops have determined the following: (i) agrobiodiversity may be lost (Hammer et al. 1996; Teklu and Hammer 2006) or conserved (Mekbib 2008; Bezançon et al. 2009); (ii) farmers may conserve agrobiodiversity to manage climate uncertainty (Orozco-Ramírez and Astier 2017; Fenzi et al. 2015), diversify their diets (Ortega-Paczka 1973), obtain profits and fulfill traditional uses (Rice 2007; Wang et al. 2017); (iii) in situ populations may contain higher diversity than ex situ accessions (Yang et al. 2005; Barry et al. 2008; Deu et al. 2010; Sun et al. 2012; Liu et al. 2017); and (iv) ex situ accessions may experience drift or inbreeding during regenerations (Parzies et al. 2000; Gómez et al. 2005). However, while comparing the genetic diversity of ex situ landrace accessions with landrace samples collected later in the same locations may provide insights about the outcomes of the conservation strategies, it does not allow to pinpoint evolutionary processes ongoing in situ.

Instead, comparing the same seed lots after the same time period of ex situ and in situ conservation may unlock the full potential of longitudinal studies to assess the extent of conserved and evolving genomic regions in any germplasm collection. This approach is supported by the genomic revolution, that has increased the power to detect genetic polymorphisms in an ever-increasing number of samples (Wambugu et al. 2018) but it is challenged by the limited capacity to identify seed lots for direct comparisons. Race-based classification systems used in the scientific community seldom coincide with farmers’ landrace-based classification and seed lot-based management: for example, scientists may classify seed lots farmers call *criollo* (local) or *blanco* (white) into various races. Thus, a thorough interaction with farmer communities is required to track down the same seed lots across generations. In this study, races are considered “groups of related individuals with enough characteristics in common to permit their recognition” (Anderson and Cutler 1942). Landraces refer to “dynamic population(s) of cultivated plants with a historical origin, distinct identity […] locally adapted and associated with traditional farming systems” (Camacho Villa et al. 2005). Seed lots refer to “all the seeds […] selected by a farmer and planted throughout a specific cultivation cycle, as well as the direct descendants of these seeds” (Louette 1994).

Mexico is an optimal location to undertake a longitudinal comparison of ex situ and in situ conservation. As the center of origin and one of the centers of diversification of maize—the world’s second most important crop in terms of cultivated area and production volume (FAO 2020)—Mexico has contributed significantly to genebank collections and currently maintains important agrobiodiversity reserves in situ. Mexican farmers have harnessed maize diversity through management decisions that take into account a complex interplay between environmental and cultural factors (Pressoir and Berthaud 2004a, 2004b; Perales et al. 2005; Brush and Perales 2007; Orozco-Ramírez et al. 2016). At present, native Mexican maize landraces represent 59 of the 219 maize races designated and characterized in Latin America (Sanchez et al. 2000), forming two of the four main diversity groups identified among New World maize populations (Vigouroux et al. 2008).

The introduction of improved varieties and hybrids following the Green Revolution combined with agricultural policies driving market integration since the 1990s have impacted the Mexican maize cultivation system (Eakin et al. 2018). Nowadays, farmers continue to cultivate maize landraces due to their flavor, quality for special preparations, diverse uses and farmer’s personal attachment to their landrace, encouraged in some cases by price premiums in urban markets that foster an enabling environment for maize landrace cultivation (Lazos and Chauvet 2012; McLean-Rodríguez et al. 2019). However, maize landraces have become in general less abundant in the fields (Ortega-Paczka 2003; CONABIO 2011). Furthermore, the relative importance of the different maize landraces has shifted (Perales et al. 2003a; Arias et al. 2007; Orozco-Ramírez and Astier 2017; Fenzi et al. 2015) because there are different incentives and disincentives for the conservation of each landrace (Perales et al. 2003b). However, the effect of ex situ and in situ conservation in maize landraces has only been compared in a few studies (Soleri and Smith 1995; Rice et al. 2006).

In this study, we report a 50-year longitudinal comparison of the genetic diversity of a group of seed lots of multiple maize landraces from the state of Morelos, Mexico. By tracing back to the families who donated the samples to the International Maize and Wheat Improvement Center (CIMMYT) Germplasm Bank in 1967 using the collection’s unique passport information, we were able to identify and resample in situ the same seed lots from which ex situ samples originated. Previously, we explored the socioeconomic factors driving the conservation of these landraces (McLean-Rodríguez et al. 2019). Here, we used genome-sequencing to genotype in situ and ex situ samples with thousands of single nucleotide polymorphism (SNP) markers. We combined this characterization with farmers’ interviews to capture the drivers of maize selection in situ. Our aim was to understand if and how the genome-wide and locus-specific genetic diversity of these seed lots changed in farmers’ fields. We hypothesized that evolutionary processes modified (i) the amount and (ii) overall distribution of genetic diversity of in situ seed lots, (iii) differentiated them from samples conserved ex situ and (iv) altered the allele frequencies of in situ seed lots at specific loci. We discuss the implications of our findings to improve the effectiveness of conservation strategies in genebanks and in farmers’ fields.

## Materials and methods

### Plant materials

This longitudinal study started with a collection of 93 maize landrace samples (henceforward, “ex situ samples*”*) donated by 66 families from the state of Morelos, Mexico, in 1967 and conserved ex situ as accessions at the CIMMYT Germplasm Bank (Supplementary Information 1). The collection was originally established to characterize the morphology, distribution and diversity of *Ancho*, a wide-kernel, high-starch landrace native to Morelos state. The collection also included other local landraces. Dr. Ángel Kato-Yamakake, a researcher at the CIMMYT Germplasm Bank at that time, collected the samples during 12 field days between December 1966 and January 1967 from farmers who agreed to donate ears from their visible harvest piles. In the metadata, Dr. Kato-Yamakake included the name of the farmer and the location or village where the sample was collected, its local common name, and the number of donated ears, later adding photographs of representative ears from each sample. These photographs were used in 2013 by Dr. Rafael Ortega-Paczka (Chapingo University) to verify the landrace classifications in the passport data (Ortega-Paczka, personal communication). The collection report (Kato 1967) was used in 2017 to trace back to the 66 families who had donated the samples to the germplasm bank (McLean-Rodríguez et al. 2019).

**Fig. 1:**
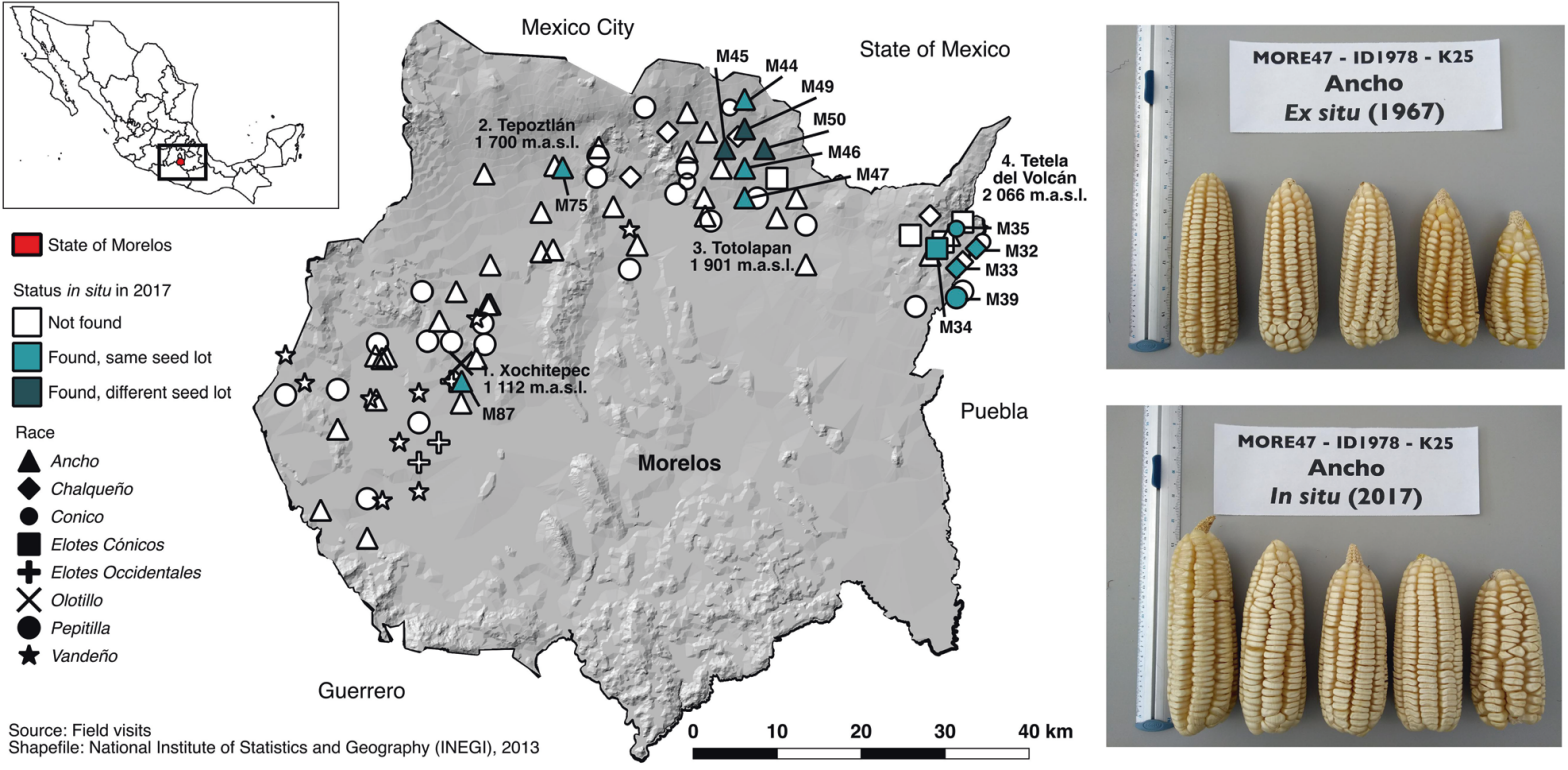
Geographical distribution of the 93 ex situ samples collected in 1967 in Morelos state, Mexico (Kato 1967), with the 13 samples collected in situ for the genetic comparison with their ex situ counterparts in 2017. Colors and shapes represent each sample’s status in 2017 and the races they represent, according to the legend. Text labels indicate the municipalities where in situ samples were collected in 2017 and samples’ IDs. Pictures illustrate representative ears for a pair of ex situ and in situ *Ancho* samples.

In 2017, 13 samples (henceforward “in situ samples”) were collected in Morelos state from those 12 families who were still cultivating landrace maize, 50 years after the original collection. Ten of these samples were collected from families who were still growing the same seed lots from the original 1967 collection (McLean-Rodríguez et al. 2019). The remaining samples were collected from families who were cultivating the same landrace as they were in 1967, but who had changed their seed lot at some point during this period. Based on an independent dataset generated by the Seeds of Discovery (SeeD) initiative (Pixley et al. 2018), the 13 samples included in this study contained ~83.1% of the alleles present in the original 1967 collection (Supplementary Information 2). The 13 samples collected in 2017 (same and different seed lots together) came from 12 families from four municipalities in Morelos, located 22–60 km apart from each other (42–110 km by roads): Xochitepec (1112 m above sea level [m.a.s.l], 1 sample), Tepoztlán (1700 m.a.s.l., 1 sample), Totolapan (1901 m.a.s.l., 6 samples) and Tetela del Volcán (2066 m.a.s.l., 5 samples) (Fig. 1). In terms of races, the eight samples from Xochitepec, Tepoztlán, and Totolapan were classified as *Ancho*, including those of different seed lots. Other samples, all from Tetela del Volcán, were classified as *Chalqueño*, *Cónico*, *Pepitilla*, and *Elotes Cónicos*. These races showed consistent phenotypic differences among them. Between six and 28 ears were included in each in situ sample to replicate the sample size of the original ex situ collection. Seeds of all ears per in situ sample were combined into one bulk, following the germplasm bank practices of the 1960s. The full set for this study comprised 26 samples grouped in 13 pairs, each with an in situ sample of a farmer-conserved seed lot and its corresponding ex situ*-*conserved counterpart (Table 1).

**Table 1:** Descriptors of the 13 ex situ and in situ sample pairs.

Passport information (CIMMYT Germplasm Bank)							Farmers’ interviews (2017)					
ID	Common name	Race^a^		Sample size (ears)	Mun^b^	Ex situ increments	Estimated surface (ha)		Distance to other maize plot (m)	Intercrop	Surrounding crops	Commercial production^c^
		Primary	Secondary				1967	2017				
Same seed lot												
M32	*Criollo*	*Chalqueño*	–	20	4	1977	3–4	1–1.5	50	Beans	Maize, beans	–
M33	*Criollo*	*Chalqueño*	*Pepitilla*	22	4	1977	3	0.5	150	Maize (*Ancho* and *Negro*)^d^	Pastures, maize	–
M34	*Negro*	*Elotes Cónicos*	*Chalqueño*	28	4	1977	3	0.5	150	Maize (*Ancho* and *Criollo*)^d^	Pastures, maize	–
M35	*Criollo/delgado*	*Cónico*	*Chalqueño*	24	4	1977	NA^e^	2.5–3	200	Avocado trees	Avocado trees, maize	–
M39	*Delgado*	*Pepitilla*	*Cónico*	6	4	1977	NA^e^	NA^e^	5	–	Maize	–
M44	*Ancho*	*Ancho*	*Cónico*	20	3	1977, 2006	0.6	0.1	50	–	Tomato, maize	–
M46	*Ancho*	*Ancho 7*	–	20	3	1977, 2017	3	2	100	Maize (hybrid barrier)	Maize	Yes
M47	*Ancho*	*Ancho 6*	–	16	3	1977	6	1.5	150	–	Nopal, maize	Yes
M75	*Ancho*	*Ancho*	*Bolita*	20	2	1977	NA^e^	1.5	10	Maize (*Pepitilla* and hybrid)	Maize	Yes
M87	*Ancho*	*Ancho*	–	20	1	1974	2.5–3	0.5	200	–	Citrus trees, maize	–
Different seed lot^f^												
M45	*Ancho*	*Ancho*	*Pepitilla*	20	3	1977	10–15	1–3	100	–	Nopal, maize	Yes
M49	*Ancho*	*Ancho 7*	–	22	3	1977	1	2	50	–	Nopal, maize	Yes
M50	*Ancho*	*Ancho 6*	–	20	3	1977	5–10	20	100	–	Nopal, maize	Yes

Sources: CIMMYT Germplasm Bank database; Kato (1967); McLean-Rodríguez et al. (2019).

^a^Race classification by Dr. Rafael Ortega-Paczka (Chapingo University) in 2013, based on the original photographs of each sample. Numbers 5–9 refer to the degree of purity (lack of influence from other established races), from lowest to highest.

^b^Municipalities: (1) Xochitepec, (2) Tepoztlán, (3) Totolapan, (4) Tetela del Volcán.

^c^In addition to household consumption.

^d^Seed lots cultivated by the same family.

^e^Farmers do not know or cannot recall the approximate maize surface cultivated in their families two generations ago/do not know the area currently cultivated.

^f^Different seed lot origins: MORE45, Market in Cuautla, Morelos, 2006; MORE49, Neighbor (the owner of MORE50) from Totolapan, Morelos, 2012; MORE50, Father-in-law from Totolapan, Morelos, 1966.

### Farmers’ interviews and focus group discussions

Farmers who conserved the seed lots found in situ were requested to describe whether other maize landraces or improved varieties were cultivated in the same or nearby plots to identify immediate sources of gene flow (Table 1), the traits they looked for when selecting seed for the next cycle, and when and how seed selection took place (Supplementary Information 3). In addition, defining traits of each landrace were identified through focus group discussions with other farmers in each of the 19 municipalities where the 93 ex situ samples were collected. Based on the photographs of ex situ samples collected in each municipality in 1967, participants listed all the landraces that they recalled were being cultivated at that time and all landraces and improved varieties cultivated in their municipality in 2017. Participants were also requested to list the positive and negative traits associated with each landrace in the list. Further details can be found in McLean-Rodríguez et al. (2019).

### Genotyping

For each of the 26 samples in the set, 10 individual seedlings were genotyped. This number was chosen to detect heterozygosity at any given diploid locus with a binomial probability greater than 0.99. Genomic DNA was extracted from fresh leaves of 7-day-old seedlings using a GenElute^TM^ Plant Genomic DNA Miniprep Kit (Sigma-Aldrich, Saint Louis, MO, USA) following the manufacturer’s instructions. Genomic DNA was quantified in a Qubit^TM^ 3.0 Fluorometer (Thermo Fisher Scientific Inc., Waltham, MA, USA) using a Qubit dsDNA BR Assay Kit. Double-digestion restriction-site associated DNA (ddRAD) markers (Baird et al. 2008) were derived from sequencing on a HiSeq2500 platform (Illumina, San Diego, CA, USA) at IGATech (Udine, Italy). ddRAD libraries were produced using an IGATech custom protocol, with minor modifications according to Peterson et al. (2012). An in silico analysis of the B73 Reference Genome V4 was used to select the best combination of two restriction enzymes and the best fragment size distribution to obtain the desired number of loci. *Pstl* and *EcoRI* were selected and used in this experiment. Raw Illumina reads were de-multiplexed using the Stacks v2.0 *process_radtags* utility (Catchen et al. 2013). Raw reads are available at the European Nucleotide Archive (https://www.ebi.ac.uk/=) under study code PRJEB41410. Uniquely aligned reads (mapping quality >4) were realigned to the reference genome with the BWA-MEM package using default parameters (Li and Durbin 2009). SNPs from aligned reads were detected with Stacks v2.0 *gstacks* program using default parameters (Catchen et al. 2013). Detected loci were filtered with Stacks v2.0 *populations* program, setting option –r to 0.7. Hence, the resulting SNP dataset contained all markers that featured less than 30% missing allele calls (N) for the entire set of 260 genotypes (seedlings). No imputation or additional filtering was performed. For further analyses, genotypes were divided into subgroups by samples, pairs, races, municipalities or conservation strategies (ex situ and in situ).

### Genetic diversity

To compare the genetic diversity between ex situ and in situ samples, polymorphism count, observed and expected heterozygosity and minor allele frequencies (MAF) were estimated in the R environment (R Core Team 2017) with the *R/adegenet* package (Jombart 2008). Minor alleles were classified as MAF < 1% (rare), MAF 1–5% (low frequency) or MAF > 5% (common). A site-frequency spectrum (SFS) comparison was implemented with the *R/vcf2sfs* script suite (Liu et al. 2018). The occurrence and frequency of private alleles in ex situ and in situ samples were examined using the *private_alleles* function from *R/poppr*, version 2.8.1 (Kamvar et al. 2014). Statistically significant differences in means, proportions and distributions were identified using *t* test, chi-square tests and Kolmogorov–Smirnov test, respectively.

### Variance distribution

To represent the relationships among all samples in the set, a neighbor joining phylogeny was built using the Euclidean distance matrix from the *nj* function from *R/ape* (Paradis et al. 2004). To summarize the total variance in the set, a principal component analysis (PCA) was performed using the *prcomp* function in *R/stats*. The relationship between ex situ and in situ samples and between samples of different pairs, races and municipalities were compared to explore if these factors were associated with any structure in the molecular data. To identify the main sources of variance in the set, analyses of molecular variance (AMOVA) were performed using the *poppr.amova* function (*ade4* implementation) from *R/poppr* (Excoffier et al. 1992), grouping the seedlings by samples, pairs, ex situ or in situ conservation strategy, races and municipalities. To assess differentiation between samples, pairwise *F*_st_ were estimated using *R/hierfstat* with the Weir and Cockerham estimator (Weir and Cockerham 1984; Goudet 2005). Confidence intervals (95%, 100 bootstraps) were estimated using the *boost.ppfst* function.

### Population structure

In order to identify and describe the population structure present in the set based on the molecular data, a discriminant analysis of the principal components (DAPC) was performed with *R/adegenet* (Jombart et al. 2010). As a non-parametric method, the DAPC is more robust than model-based population structure inference methods to detect population limits (Linck and Battey 2019). A k-means clustering procedure was implemented with the *find.cluster* function to identify the optimal number of clusters and the group memberships to input into the DAPC, based on the Bayesian Information Criterion. The *optim.a.score* function was used to choose the optimum number of PCs to retain. To identify the most important markers contributing to between-group variance, the variable loadings of the main discriminant functions were plotted against their physical position. Posterior group assignments were plotted to compare the distribution of ex situ and in situ seedlings in the *k* groups.

The pattern of genome-wide linkage disequilibrium in the set was studied as a further indicator of genetic structure. Pairwise LD was calculated for all markers per chromosome using *R/LDheatmap* (Shin et al. 2006). *r*^*2*^ was selected over *D*′ as it accounts for differences in MAF between loci. To reduce computational time, only the SNPs with MAF > 10% were included. LD decay by chromosome as a function of physical distances was estimated based on the Hill and Weir equation (Hill and Weir 1988). A threshold of *r*^*2*^ = 0.1 was imposed to compare rates of LD decay between chromosomes. To evaluate the evolution of long-range LD along each chromosome, average pairwise *r*^*2*^ for all surrounding markers within a ±10 Mb window was estimated for each SNP. The resulting LD was plotted against physical positions, averaging values over a sliding window of 5% of each chromosome’s markers.

### Genomic signatures of selection

Candidate loci under selection between ex situ and in situ paired samples were identified using two outlier detection methods implemented in *R/OutFLANK* (Whitlock and Lotterhos 2015) and BayeScan v2.1 (Foll and Gaggiotti 2008). OutFLANK uses a procedure based on an inferred distribution of neutral *F*_st_ values that is used to assign *q* values to each locus to identify *F*_st_ outliers that may be due to directional selection. BayeScan implements an island model based on the multinomial Dirichlet distribution in which subpopulation-specific *F*_st_ represents the allele frequency differences between subpopulations and the common gene pool. *F*_st_ coefficients are divided into subpopulation-specific components (beta) and locus-specific components (alpha). Loci with a locus-specific component significantly different from zero are considered to be under selection. Additional tests between ex situ and in situ samples from each of the races and municipalities represented in the set were implemented in BayeScan, to further explore the results. Both outlier detection methods were implemented on a reduced dataset filtered for SNPs with a MAF below 1% using default parameters. Significant loci were identified in both methods using a false discovery rate (*q* value) of 5%.

### Annotation of candidate genes and overlap with reported QTLs

To identify gene functions potentially associated with population structure and selection-related SNPs, annotations were searched in the Zm00001d.2 Filtered Gene Set from MaizeGDB Maize B73 RefGen_v4 Genome browser: https://maizegdb.org/gbrowse/maize_v4 (Portwood et al. 2019). Gene models and associated protein coding genes and gene functions were searched in regions within ±1 Mb from markers with the highest DAPC loadings and significant markers identified with the two outlier detection methods. A literature search was performed for QTL associated with traits under farmers’ selection, from which QTL coinciding with selection-related SNPs were identified. When not reported, flanking markers’ physical positions were obtained from MaizeGDB Probe/Molecular Marker Data: https://maizegdb.org/data_center/marker.

## Results

### Genetic diversity

After filtering for quality, 74,739 genome-wide SNPs were retained from 260 genotyped seedlings belonging to the 13 ex situ samples and 13 in situ samples in the set. SNPs were distributed along the genome at an average density of 35 SNP/Mbp (a range of 1–170 SNP/Mbp) (Supplementary Information 4). Forty-six percent of the SNPs in the set were rare (MAF < 1%), 31% of low frequency (MAF 1–5%) and 23% were common (MAF > 5%). Ex situ and in situ heterozygosity levels did not vary with the number of sampled ears (Supplementary Information 5).

Diversity indicators showed that in situ samples were less diverse than ex situ samples, and this was due to a reduction in the number of alleles, but not in expected or observed heterozygosity (Table 2). In situ samples contained 3.1% fewer SNPs than ex situ samples. Expected heterozygosity compared to observed heterozygosity suggested some degree of inbreeding, but this level remained constant over time. Ex situ and in situ samples showed small significant differences in their SFS (Supplementary Information 6). However, rare and low frequency alleles were predominant in both ex situ and in situ samples. Twenty and 17% of the SNPs reported private alleles in ex situ and in situ samples, respectively, but most of these private alleles had rare or low frequencies (MAF ≤ 5%). Comparisons between ex situ and in situ samples in the same pairs did not suggest any pattern by race or municipality (Supplementary Information 5). However, they showed that three pairs from different municipalities and races (M35, *Cónico* from Tetela del Volcán; M46, *Ancho* from Totolapan; M87, *Ancho* from Xochitepec) showed significantly less polymorphism in situ. Other pairs showed significantly more polymorphism in situ or no significant differences between ex situ and in situ samples. Pairs M35 and M87 were the only ones showing significantly lower expected and observed heterozygosity in situ than ex situ.

**Table 2:** Genetic diversity indicators for ex situ and in situ samples.

Indicator	Ex situ samples (*n* = 13)^a^	In situ samples (*n* = 13)^a^	*P* value
Number of polymorphic SNPs^b^	61,789 (82.67%)	59,510 (79.62%)	<0.0000
Divided by MAF frequency^c^			
<1%	20,496 (27.42%)	19,087 (25.54%)	<0.0000
1–5%	23,569 (31.54%)	22,769 (30.46%)	
>5%	17,724 (23.71%)	17,654 (23.62%)	
He	0.0781	0.0770	0.0720
Ho	0.0561	0.0562	0.7715
Private alleles^b^	15,229 (20.38%)	12,950 (17.33%)	<0.0000
Divided by MAF frequency			
<1%	10,341 (13.84%)	8,551 (11.44%)	0.0013
1–5%	4,815 (6.44%)	4,316 (5.77%)	
>5%	73 (0.1%)	83 (0.11%)	

^a^Each sample combines the genotypes of ten single seedlings, individually genotyped.

^b^From a total of 74,739 SNPs in the complete dataset, after monomorphic SNPs for each of the two subgroups were removed. Percentages shown in parentheses are based on this figure.

^c^MAF per SNP were estimated based on the number of alleles present for that marker in the corresponding 130 genotypes of each subgroup.

### Variance distribution

**Fig. 2:**
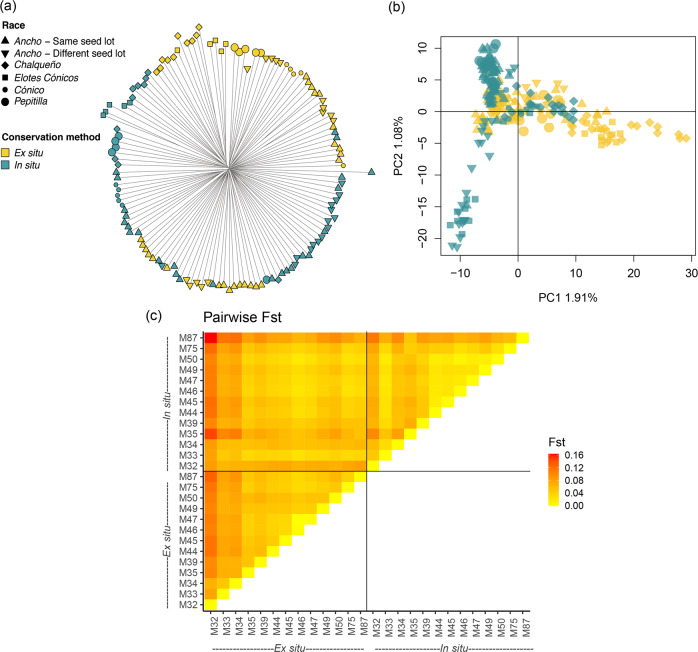
Genetic diversity within the set. **a** Neighbor joining phylogeny representing the relationship between ex situ and in situ samples. For each sample, five random seedlings are represented. **b** Principal Component Analysis (PCA) including all samples. PC1 and PC2 are shown on the *x* and *y-*axes, respectively, with the percentage of explained variance. Colors and shapes represent each sample’s conservation strategy (ex situ or in situ) and its race, respectively, according to the legend. **c** Pairwise *F*_st_ between samples. Cell colors represent *F*_st_ values according to legend. Samples are represented in the *x* and *y-*axes.

The phylogenetic analysis confirmed that in situ seed lots had not markedly diverged from samples conserved ex situ. Seedlings did not form any clearly differentiated clusters (Fig. 2a, Supplementary Information 7). Consistent with limited structure, each principal component (PC) in the PCA contributed only marginally to explain the total variance (1.91% and 1.08% for the first two PCs). Ex situ and in situ samples overlapped in the PC space (Fig. 2b). The first PC partially distinguished most pairs of *Chalqueño, Elotes Cónicos, Cónico* and *Pepitilla* from Tetela del Volcán from pairs of *Ancho* from the other municipalities, while the second PC partially distinguished three in situ samples (M35, *Cónico* from Tetela del Volcán; M75, *Ancho* from Tepoztlán; M87, *Ancho* from Xochitepec) from other samples in the set. The AMOVA showed that conservation strategy (ex situ or in situ) explained only 0.7% of the variance (Supplementary Information 8). Instead, most of the variance was found among individual seedlings. All of the alternative groups tested (samples, pairs, races or municipalities) also explained only a small share of the variance.

Pairwise *F*_st_ confirmed there was little to moderate genetic distance between samples (Fig. 2c, Supplementary Information 9) (Hartl and Clark 1997). The greatest genetic distance was observed between ex situ sample M32 (*Cónico* from Tetela del Volcán) and in situ sample M87 (*Ancho* from Xochitepec) (*F*_st_ = 0.164). The smallest distance was observed between two undifferentiated ex situ samples from Totolapan (*F*_st_ ~ 0). Excluding this case, distances were smaller between in situ samples than between ex situ samples or between ex situ and in situ samples (Fig. 2c). Distances ranged between 0.021 and 0.085 for ex situ and in situ sample pairs of the same seed lot, and between 0.041 and 0.045 for sample pairs of different seed lots (M45, M49 and M50). An *F*_st_-based phylogeny coincided with the SNP-based phylogeny, further highlighting the differentiation of samples from Tetela del Volcán (*Chalqueño* M32 and M33, and *Elotes Cónicos* M34), and Xochitepec (*Ancho* M87) from the rest of the samples (Supplementary Information 10).

### Population structure

**Fig. 3:**
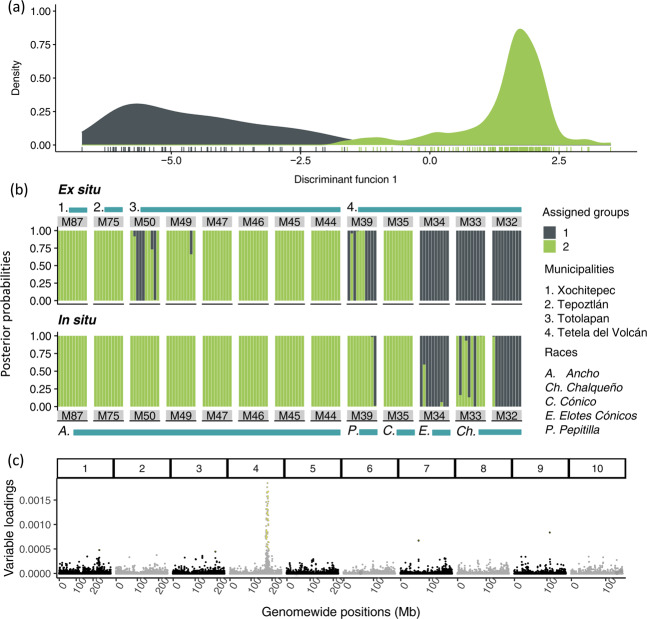
Discriminant analysis of principal components (DAPC) including all samples, with seedlings assigned into *k* = 2 groups. **a** Scatter plot of the discriminant function with values reported on the *x*-axis. Individual seedlings are represented by tick marks below the estimated distributions. **b** Posterior group assignments for *k* = 2 groups. Ex situ and in situ samples are shown in the upper and lower panels, respectively. Each vertical bar represents an individual seedling, with colors corresponding to the two assigned genetic groups, according to the legend. Numbers and color bars are reported above and below the graph indicating the municipalities and race for each sample. **c** Variable loadings for the discriminant function, plotted by genomic position (*x*-axis) in shadings alternating by chromosome. *Y*-axis shows the variable loadings with the highest loading markers highlighted in yellow.

**Fig. 4:**
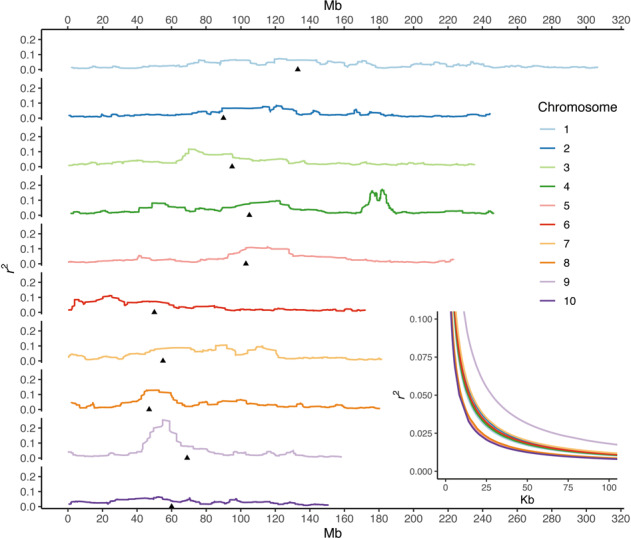
Genomic linkage disequilibrium (LD) within the set. The insert bottom right shows LD decay by chromosome. The large panel shows the average *r*^*2*^ (*y-*axis) plotted against physical distance (*x*-axis) by chromosomes. Chromosomes are colored according to legend. Black triangles represent centromere positions.

All samples in the set were identified as belonging to a single genetic population and the limited structure present in ex situ samples has been, in general, conserved in situ. A k-mean clustering procedure consistently indicated one as the optimum cluster number to describe the set (Supplementary Information 11). The second most likely grouping, made of two clusters, was retained to identify the main source of the limited structure present (Fig. 3a). Based on a-score optimization, 15 PCs were retained in the DAPC for *k* = 2. Within ex situ samples, posterior group assignments distinguished samples M32, M33 and M34 as well as seedlings from samples M50 and M39 (group 1) from the rest (group 2) (Fig. 3b). Most samples assigned to group 1 belonged to the municipality of Tetela del Volcán and to races other than *Ancho*, while samples assigned to group 2 belonged to municipalities other than Tetela del Volcán and to the *Ancho* race. This structure was mostly conserved in situ. Within in situ samples, samples M34 and M32 were also assigned to group 1. However, the contribution of group 1 was less evident in samples M33 and 39 and no longer evident in sample M50. Markers with the highest loading maximizing the variance between group 1 and 2 were located in chromosomes 1, 3, 7, and 9, but mainly in a region between 170.75 and 185.95 Mbp in chromosome 4 (Fig. 3c). This region, in addition to the centromeres and peri-centromeres, was also identified as having a relatively high LD (Fig. 4). Genome-wide LD within the set was low and represented fast rates of decay: the *r*^*2*^ declined to 0.1 at an average distance of 6.3 kbp (Fig. 4, Supplementary Information 12).

### Traits under farmers’ selection

Farmers reported during interviews that ears were their unit of selection and, as a result, they would favor traits of overall ear fitness regardless of their seed lot’s landrace. Selection took place invariably after the harvest in farmers’ houses or sheds, where they selected the best ears from the harvest pile, while removing the husk or before shelling. Preferred traits included large, heavy, full, healthy ears, with uniform kernel coverage and no visible mechanical or disease damage (Table 3). Following this process, farmers did not select for or against any favorable or unfavorable plant trait. Nonetheless, farmers favored certain ear traits for specific landraces/races. These traits included wide and white kernels and eight kernel rows per ear for *Ancho* (Fig. 1), black kernels for *Elotes Cónico*, and spiky kernels for *Pepitilla*. Focus groups discussions revealed that these landrace-specific traits were related to features that most farmers valued in their landraces, such as their use in special preparations (including *pozole*, a hominy soup prepared with *Ancho*, and *antojitos*, tortilla-based dishes frequently prepared with *Elotes Cónico*) and the higher price these landraces can obtain in the market for these special uses compared to improved maize varieties (Supplementary Information 13).

**Table 3:** Traits farmers use to select seed for the next cycle, by race.

Trait	*Ancho*	*Chalqueño*	*Cónicos*	*Elotes Cónicos*	*Pepitilla*
Ear					
Full	x	x	x	x	x
Healthy	x				
Heavy	x				
Large	x	x	x	x	x
Well-developed	x	x	x	x	x
Cob					
Thin	x	x	x	x	x
Kernel rows					
8 per ear	x				
Straight	x				
Uniform	x				x
Kernels					
Black				x	
Spiky					x
Wide	x				

### Genomic signatures of selection

The two alternative outlier detection methods jointly identified eight loci under section when comparing ex situ and in situ samples (Table 4). OutFLANK reported 168 markers as outliers when comparing all ex situ samples with all in situ samples (Supplementary information 14). BayeScan reported ten outlier loci, four of these when comparing all ex situ samples with all in situ samples, and six when focusing on specific races and municipalities (Supplementary Information 15). Eight outliers located at 224.17 Mbp in chromosome 3, 245.09 Mbp in chromosome 4, 42.79 Mbp in chromosome 5, 52.20 and 53.28 and 58.48 Mbp in chromosome 6 and 135.91 and 147.74 Mbp in chromosome 10 coincided between the two methods. In both analyses, the strongest signal was observed at the telomeric end of chromosome 10.

**Table 4:** Loci displaying evidence of selection between ex situ and in situ samples identified with OutFLANK and BayeScan.

Chr	Pos (Mb)	OutFLANK			BayeScan		
		He	*F*_st_	*q* value	*q* value	Alpha	Sample subset
**3**	224.1651	0.4625	0.0782	0.0461	0.0090	2.2077	Ancho–Tepoztlán (1 pair)
**4**	245.0859	0.1665	0.1068	0.0106	0.0258	1.9789	Ancho–All municipalities (8 pairs)
**5**	42.7926	0.2367	0.1961	0.0001	0.0314	1.7899	All races–All municipalities (13 pairs)
**6**	52.2048	0.4642	0.1363	0.0026	0.0381	1.7628	Ancho–All municipalities (8 pairs)
**6**	53.2826	0.4424	0.0930	0.0259	0.0015	1.9752	Ancho–Totolapan (6 pairs)
**6**	58.4830	0.1606	0.1486	0.0017	0.0452	1.8704	All races–All municipalities (13 pairs)
**10**	135.9072	0.2076	0.1743	0.0005	0.0197	1.8473	All races–All municipalities (13 pairs)
**10**	147.7400	0.4875	0.4700	0.0000	0.0000	2.7091	All races–All municipalities (13 pairs)

## Discussion

### Comparing conservation strategies

The comparison of molecular diversity across maize seed lots highlighted contrasting effects of conservation strategies on landraces’ allele pools at the genome-wide and locus-specific levels. While genome-wide diversity was overall similar between ex situ and in situ samples, locus-specific changes seem to have occurred in seed lots in situ. Over time, farmers have been continually favoring specific ear traits through mass selection and disregarding whole plant traits (Table 3). Farmers’ criteria and methods for mass selection based exclusively on ear characteristics are consistent with those reported elsewhere in Mexico (Louette and Smale 2000). For some traits, including kernel width in *Ancho*, selection intensified in Morelos since the 1970s following market pressures emerging in the late twentieth century (Perales et al. 2003b). Such selective pressures in a background of fast LD decay due to a highly admixed ancestry (Fig. 4) might explain differentiation at single loci.

At a genome-wide level, our study shows that in situ seed lots had not significantly differentiated from their ex situ counterparts. In situ samples conserved the same diversity as ex situ samples in terms of expected heterozygosity and, while a reduction in polymorphism was observed, only three out of 13 pairs contributed to this reduction (Supplementary Information 5). Moreover, pairwise genetic distances were lower among in situ samples than among ex situ samples, suggesting some degree of allele flow over time (Fig. 2c, Supplementary Information 9 and 10). These results coincided with a previous longitudinal study focusing on maize landraces and relying on a small set of SSR markers (Rice et al. 2006). In our study, the smaller area cultivated with maize over time and the proximity of neighboring maize plots, as described by the farmers themselves, may have contributed to allele flow (Table 1). Measures of physical distances between a farmer’s own versus the neighbors’ field areas and the extent of perimeters in contact, as well as the degree of synchronization in flowering times, could be used in further research to compare the rates of cross-pollination with observed changes in genetic diversity in situ (Melé et al. 2015; Bøhn et al. 2016).

Because of their low frequency, differences in the presence/absence of specific alleles are not expected to affect the adaptation capacity of maize landrace populations (Table 2, Supplementary Information 6) (van de Wouw et al. 2009). Most of the alleles that were lost or gained over time in our study (alleles private to ex situ or in situ samples, respectively) had rare or low frequency (MAF ≤ 5%). Low frequency private alleles could also reflect sampling error rather than actual shifts in allele frequencies in the population due to selection. Because ex situ samples in our study were regenerated only once or twice between 1974 and 2017 (Table 1), we assume they did not experience genetic drift during ex situ regenerations, as a previous study on the genetic integrity of maize germplasm regenerated two and three times in CIMMYT Germplasm Bank reported (Wen et al. 2011). Further research could explore whether introgressions from commercial hybrids into landraces have occurred and to determine the negative or positive implications for maize landrace in situ conservation. Introgressions from hybrids were previously detected in maize landraces outside and in their center of origin (Bitocchi et al. 2009, 2015; Rojas-Barrera et al. 2019), and may contribute to explain differentiation mechanisms ongoing in situ.

### Genetic structure across conservation strategies

The five races included in our study, originating from various elevations in the relatively small state of Morelos (Table 1), had limited influence on genetic structure (Fig. 2a, Supplementary Information 10). Previous studies on 46 of the 59 native Mexican races also found limited power in race identity to explain genetic variation (Arteaga et al. 2016), although a few locus-specific signals could distinguish accessions by their race and altitudinal origin (Caldu-Primo et al. 2017). However, races included in these studies were originally selected for maximum phenotypic contrast in terms of ear, kernel, plant and maturation traits, and altitudinal range, and hence differentiation loci may not coincide with population structure and selection-related SNPs identified in our study (Fig. 3c and Table 4). Overall, high levels of recombination between races were reflected in the observed low levels and rapid decay of genome-wide LD (Fig. 4) (Remington et al. 2001). LD patterns detected in our study reflect the higher genetic diversity of tropical landraces (Tenaillon et al. 2001; Yan et al. 2009) compared to elite germplasm from temperate regions (Ching et al. 2002). Still, since in this and other studies SNP-based allele calls are unphased, frequencies of rare haplotypes may be disproportionally affected by the uncertainty introduced during haplotype inference for *r*^2^ calculations (Slatkin 2008). The limited genetic structure present in ex situ samples was conserved over time in in situ samples (Fig. 3b). PCA and DAPC confirmed that not enough genetic changes have accumulated in 50 years for ex situ and in situ seed lots to form distinct populations (Figs. 2a, b and 3). Instead, the combination of highland-adapted samples from Tetela del Volcán with mid- and lowland-adapted samples from other municipalities (Fig. 1) was the main determinant of genetic structure in the set.

The high-loading and high-LD locus between 170.75 and 185.95 Mbp in chromosome 4 (Figs. 3c and 4) corresponds to a region of introgression from teosinte (*Zea mays* ssp. *mexicana*, maize’s closest wild relative) into maize, which contributed to the adaptation of maize landraces to the Mexican highlands (Hufford et al. 2013; Wang et al. 2017). This region corresponds to *Inv4m*, a well-characterized inversion polymorphism differentiating lowland teosinte (*Zea mays* ssp. *parviglumis*) from highland teosinte (*Zea mays* ssp. *mexicana*) (Lauter et al. 2004; Pyhäjärvi et al. 2013). Multiple annotations denoting highland teosinte’s influence were reported within this region. These included *pcna2*, involved in plant and inflorescence architecture (Studer et al. 2017); *tu1*, linked to the development of large glumes around individual kernels (Wingen et al. 2012); an annotated gene predicted to synthesize anthocyanin 5-aromatic acyltransferase, potentially involved in conferring dark leaf sheath pigmentation (Lauter et al. 2004; Paulsmeyer et al. 2018); and genes *cle24*, *phos2*, *ss5*, *o1* and *acco20*, all with potential implications with respect to plant morphology, nutrition, grain development and phytohormone biosynthesis (Gonzalez-Segovia et al. 2019) (Supplementary Information 16). Based on their suggestive functions, these annotations may determine yield quality and quantity, and may have been under indirect farmers’ selection when full, large, and well-developed ears produced in the highlands were favored (Table 3). Twenty outliers were identified with OutFLANK in this region, although this selection signal was not detected using BayeScan (Supplementary Information 14 and 15).

### Ongoing selection in farmer fields

Although at a genome-wide scale in situ samples were not different from ex situ samples, locus-specific changes were observed and may be potentially associated with farmers’ selection over the past 50 years. The two outlier detection methods found largely overlapping results (Table 4), even with differences in the final set of outliers reported (Supplementary Information 14 and 15). It is known that different outlier detection methods have different performances depending on sampling design and the genomic architecture of selection signatures (Lotterhos and Whitlock 2015; Hoban et al. 2016; Matthey-Doret and Whitlock 2019). The fact that OutFLANK detected eight out of ten BayeScan outliers reinforces the significance of the candidate loci jointly identified by the two methods.

QTL associated with ear, kernel and yield-related traits were identified from the literature as coinciding with most loci identified with the outlier detection methods (Supplementary Information 17). QTL for ear weight and kernel weight per ear co-mapped with the outlier loci in chromosome 3 (Yi et al. 2019). QTL for ear diameter (Sabadin et al. 2008), grain yield (Alves Lima et al. 2006), hundred-kernel weight, kernel number per row (Chen et al. 2016) and kernel width (Hui et al. 2015; Chen et al. 2016; Raihan et al. 2016) co-mapped with the outlier in chromosome 5. QTL for grain yield per plant co-mapped with the outliers at 52.20 and 53.28 Mbp in chromosome 6 (Su et al. 2017), while QTL for kernel width co-mapped with all outliers in chromosome 6 (Zhang et al. 2014; Chen et al. 2016) and the outlier at 135.91 Mbp in chromosome 10 (Hui et al. 2015). For the locus at 147.74 Mbp in chromosome 10 showing the strongest evidence for selection, co-locating QTL associated with ear length, kernel number per row (Huo et al. 2016), cob and kernel length (Liu et al. 2019), kernel volume, kernel weight (Zhang et al. 2014), and kernel width (Hui et al. 2015; Zhu et al. 2018) were reported.

Comparing reported co-locating QTL (Supplementary Information 16 and 17) with farmers’ selection criteria (Table 3) reinforces the functional interpretation of the identified outliers, although it is not definite proof of directional selection. This is because yield-related QTL are manifold and not completely disclosed in maize, depending also on the specific haplotypes segregating in the population used for QTL mapping (Martinez et al. 2016) and mapping in different locations depending on the recombination landscape in the segregating populations used to map QTL. Still, the explicit involvement of farmers in this research reinforces the functional interpretation of our findings. The kernel width trait offers an example of this: stronger selection for this trait may result from the combination of the relatively recent history of a market demand for wider kernels in *Ancho* with the ease of detection and high heritability of the trait (Table 3) (Perales et al. 2003b). Indeed, co-mapping QTL for kernel width were identified in all but two of the detected outliers (Supplementary information 17) and additional outliers were identified when analyzing *Ancho* samples pairs independently with BayeScan (Supplementary Information 15).

## Conclusions

Although the genome-wide genetic diversity and structure of maize landrace seed lots did not significantly change in farmers’ fields after 50 years of in situ conservation, there was evidence of directional selection in specific loci. This evidence was consistent with farmers’ ear-based mass selection criteria. Gene flow maintained or increased polymorphism in most seed lots in situ while reducing the genetic distances among them. On the other hand, reduced polymorphism was detected in some seed lots, but not to an extent that would be expected to hinder their adaptation capacity.

Our findings indicate that, after five decades, farmers can maintain the genetic diversity of their maize landrace populations, and ex situ accessions from genebanks such as CIMMYT’s are still representative of the diversity that is present in farmers’ field. These results identify the potential to strengthen the in situ conservation of landraces by reintroducing agrobiodiversity lost in the field, but still conserved in genebanks. While landraces whose market demand has increased over the past decades will very likely continue to be maintained in situ, changes might still occur in locus-specific allele frequencies that may not be adequately represented in genebanks. The appearance of new alleles or a change of frequency of historical alleles as a consequence of socioeconomic changes require periodic updating of ex situ collections through in situ resampling, to ensure they remain relevant for potential users. Further research should evaluate the phenotypic differences between ex situ and in situ populations in Morelos and elsewhere, to test if agrobiodiversity successfully conserved ex situ still expresses the phenotypes that meet farmers’ environmental, culinary, and commercial preferences and needs.

While our results are encouraging for agrobiodiversity conservation, the small number of seed lots still found in the same families in situ suggests that a genetic bottleneck might be avoided due to the special attributes of those few farmers who continue to cultivate and conserve landraces. Indeed, an important share of certain landrace seed lots has been lost over the past five decades from farmers’ fields (Perales et al. 2003b; CONABIO 2011; McLean-Rodríguez et al. 2019). However, most of the farmers contacted for this study appreciated learning that seed from their relatives had been conserved in the genebank and were eager to obtain them again. In particular, the collaborating families who were still cultivating the same seed lots of their ancestors valued that researchers had returned after decades and requested to be kept informed and involved in future studies. This underscores the underappreciated value to be gained by ex situ collections working directly with their in situ conservation partners, the new generation of young farmers and future custodians of the germplasm and culture sustaining agrobiodiversity.

## Supplementary information

Supplementary Information

## Data Availability

All raw reads are deposited at the European Nucleotide Archive (https://www.ebi.ac.uk/=) under study PRJEB41410. Allele calls used in the study are available in VCF format at 10.6084/m9.figshare.13199918. R scripts used for the analyses are stored on the GitHub page of the corresponding author at https://github.com/mdellh2o/morelosMAIZE. All accessory data are included as supplementary materials of this paper.
