# Supplementary material for: Genetic diversity and selection signatures in maize landraces compared across 50 years of in situ and ex situ conservation
Source: Heredity (Edinb). 2021 Mar 30;126(6):913–28. doi: 10.1038/s41437-021-00423-y (PMC8178342; doi:10.1038/s41437-021-00423-y)
Supplement: Supplementary file 1 — Supplementary Information [file 41437_2021_423_MOESM1_ESM.docx]

### Genetic diversity and selection signatures in maize landraces compared across 50 years of in situ and conservation

Francis Denisse McLean-Rodríguez, Denise E. Costich, Tania Carolina Camacho-Villa, Mario Enrico Pè, Matteo Dell'Acqua

# Supplementary Information 1. Passport information for the 93 samples collected in 1967 in Morelos, Mexico

| ID | Accession ID | Common name | Race^a^ | | Sample size (ears) | Municipality |
| --- | --- | --- | --- | --- | --- | --- |
|  |  |  | **Primary** | **Secondary** |  |  |
| MORE23 | 1954 | *Criollo* | *Chalqueño* | *Ancho* | 15 | Tetela del Volcán |
| MORE24 | 1955 | *Criollo* | *Ancho* | *Cónico* | 9 | Tetela del Volcán |
| MORE25 | 1956 | *Ancho* | *Ancho* | *-* | 16 | Tetela del Volcán |
| MORE26 | 1957 | *Negro* | *Elotes Cónicos 6* | *-* | 11 | Tetela del Volcán |
| MORE27 | 1958 | *Ancho* | *Ancho* | *-* | 12 | Yecapixtla |
| MORE28 | 1959 | *Delgado* | *Pepitilla* | *-* | 13 | Yecapixtla |
| MORE29 | 1960 | *Criollo negro* | *Elotes Cónicos* | *Chalqueño* | 21 | Tetela del Volcán |
| MORE30 | 1961 | *Criollo* | *Chalqueño* | *-* | 20 | Tetela del Volcán |
| MORE31 | 1962 | *Rápido / Cuarenteño* | *Cónico* | *Ancho* | 17 | Tetela del Volcán |
| MORE32* | 1963 | *Criollo* | *Chalqueño* | *-* | 20 | Tetela del Volcán |
| MORE33* | 1964 | *Criollo* | *Chalqueño* | *Pepitilla* | 22 | Tetela del Volcán |
| MORE34* | 1965 | *Negro o Pinto* | *Elotes Cónicos* | *Chalqueño* | 28 | Tetela del Volcán |
| MORE35* | 1966 | *Criollo / Delgado* | *Cónico* | *Chalqueño* | 24 | Tetela del Volcán |
| MORE36 | 1967 | *Lengua de Pájaro* | *Pepitilla 8* | *-* | 20 | Zacualpan de Amilpas |
| MORE37 | 1968 | *Delgado* | *Pepitilla 6* | *-* | 6 | Tetela del Volcán |
| MORE38 | 1969 | *Negro* | *Elotes Cónicos* | *Mezcla* | 6 | Tetela del Volcán |
| MORE39* | 1970 | *Criollo* | *Pepitilla* | *Cónico* | 6 | Tetela del Volcán |
| MORE40 | 1971 | *Ancho* | *Ancho* | *Olotillo* | 20 | Atlatlahucan |
| MORE41 | 1972 | *Criollo* | *Pepitilla 7* | *-* | 20 | Atlatlahucan |
| MORE42 | 1973 | *Ancho* | *Ancho* | *-* | 19 | Atlatlahucan |
| MORE43 | 1974 | *Ancho negro* | *Elotes Cónicos* | *Elotes Occidentales* | 19 | Atlatlahucan |
| MORE44* | 1975 | *Ancho* | *Ancho* | *Cónico* | 20 | Totolapan |
| MORE45* | 1976 | *Ancho* | *Ancho* | *Pepitilla* | 20 | Totolapan |
| MORE46* | 1977 | *Ancho* | *Ancho 7* | *-* | 20 | Totolapan |
| MORE47* | 1978 | *Ancho* | *Ancho 6* | *-* | 16 | Totolapan |
| MORE48 | 1979 | *Criollo* | *Chalqueño 6* | *-* | 8 | Totolapan |
| MORE49* | 1980 | *Ancho* | *Ancho 7* | *-* | 22 | Totolapan |
| MORE50* | 1981 | *Ancho* | *Ancho 6* | *-* | 20 | Totolapan |
| MORE51 | 1982 | *Ancho* | *Ancho* | *Pepitilla* | 20 | Tlayacapan |
| MORE52 | 1983 | *Delgado* | *Pepitilla* | *-* | 20 | Yautepec |
| MORE53 | 1984 | *Del monte* | *Cónico* | *-* | 20 | Tlalnepantla |
| MORE54 | 1985 | *Ancho* | *Ancho* | *-* | 22 | Tlalnepantla |
| MORE55 | 1986 | *Delgado* | *Pepitilla* | *-* | 22 | Tlalnepantla |
| MORE56 | 1987 | *Del monte* | *Chalqueño 6* | *-* | 20 | Tlalnepantla |
| MORE57 | 1988 | *Del monte* | *Cónico* | *-* | 20 | Tlalnepantla |
| MORE58 | 1989 | *Ancho* | *Ancho 7* | *-* | 20 | Tlalnepantla |
| MORE59 | 1990 | *Ancho* | *Ancho 6* | *-* | 20 | Tlalnepantla |
| MORE60 | 1991 | *Ancho* | *Ancho 7* | *-* | 20 | Tlayacapan |
| MORE61 | 1992 | *Delgado* | *Pepitilla* | *-* | 20 | Tlayacapan |
| MORE62 | 1993 | *Ancho* | *Ancho* | *-* | 20 | Tlayacapan |
| MORE63 | 1994 | *Delgado* | *Pepitilla* | *-* | 20 | Yautepec |
| MORE64 | 1995 | *Ancho* | *Ancho* | *Pepitilla* | 20 | Yautepec |
| MORE65 | 1996 | *Delgado* | *Pepitilla* | *-* | 20 | Tepoztlán |
| MORE66 | 1997 | *Ancho* | *Ancho* | *Vandeño* | 20 | Tepoztlán |
| MORE67 | 1998 | *Amarillo* | *Chalqueño* | *Vandeño* | 20 | Tepoztlán |
| MORE68 | 1999 | *Delgado* | *Pepitilla* | *-* | 20 | Yautepec |
| MORE69 | 2000 | *Tehuacán* | *Vandeño* | *-* | 22 | Yautepec |
| MORE70 | 2001 | *Ancho* | *Ancho* | *Pepitilla* | 20 | Jiutepec |
| MORE71 | 2002 | *Ancho* | *Ancho* | *Vandeño* | 20 | Jiutepec |
| MORE72 | 2003 | *Ancho negro* | *Ancho* | *Elotes Cónicos* | 20 | Jiutepec |
| MORE73 | 2004 | *Ancho* | *Ancho 7* | *-* | 20 | Cuernavaca |
| MORE74 | 2005 | *Ancho* | *Ancho 7* | *-* | 20 | Tepoztlán |
| MORE75* | 2006 | *Ancho* | *Ancho* | *Bolita* | 20 | Tepoztlán |
| MORE76 | 2007 | *Delgado* | *Pepitilla* | *Chalqueño* | 20 | Tepoztlán |
| MORE77 | 2008 | *Ancho* | *Ancho* | *Olotillo* | 20 | Tepoztlán |
| MORE78 | 2009 | *Ancho* | *Ancho* | *-* | 20 | Temixco |
| MORE79 | 2010 | *Ancho* | *Ancho* | *-* | 20 | Temixco |
| MORE80 | 2011 | *Ancho* | *Ancho* | *-* | 20 | Temixco |
| MORE81 | 2012 | *Delgado* | *Pepitilla* | *-* | 20 | Xochitepec |
| MORE82 | 2013 | *Criollo* | *Pepitilla* | *Vandeño* | 30 | Xochitepec |
| MORE83 | 2014 | *Ancho de color* | *Vandeño* | *Pepitilla* | 20 | Xochitepec |
| MORE84 | 2015 | *Ancho* | *Ancho* | *Pepitilla* | 26 | Xochitepec |
| MORE85 | 2016 | *Ancho* | *Ancho* | *-* | 23 | Xochitepec |
| MORE86 | 2017 | *Itzihuine amarillo* | *Chalqueño* | *-* | 20 | Xochitepec |
| MORE87* | 2018 | *Ancho* | *Ancho* | *-* | 20 | Xochitepec |
| MORE88 | 2019 | *Negro* | *Olotillo* | *Elotes Occidentales* | 20 | Xochitepec |
| MORE89 | 2020 | *Colorado* | *Elotes Occidentales* | *-* | 22 | Xochitepec |
| MORE90 | 2021 | *Blanco* | *Pepitilla* | *Vandeño* | 26 | Xochitepec |
| MORE91 | 2022 | *Delgado* | *Pepitilla* | *-* | 20 | Miacatlán |
| MORE92 | 2023 | *Ancho* | *Ancho* | *Pepitilla* | 30 | Temixco |
| MORE93 | 2024 | *Ancho* | *Ancho* | *Pepitilla* | 30 | Temixco |
| MORE94K | 2025 | *Delgado* | *Pepitilla* | *-* | 20 | Temixco |
| MORE95 | 2026 | *Ancho* | *Ancho* | *Pepitilla* | 20 | Miacatlán |
| MORE96 | 2027 | *Ancho* | *Ancho* | *Pepitilla* | 21 | Miacatlán |
| MORE97 | 2028 | *Delgado* | *Pepitilla* | *-* | 20 | Miacatlán |
| MORE98 | 2029 | *Ancho* | *Ancho* | *Olotillo* | 20 | Miacatlán |
| MORE99 | 2030 | *Ancho* | *Ancho* | *Vandeño* | 20 | Mazatepec |
| MORE100 | 2031 | *Tehuacán* | *Vandeño* | *Ancho* | 20 | Mazatepec |
| MORE101 | 2032 | *Ancho* | *Ancho* | *Olotillo* | 20 | Tetecala |
| MORE102 | 2033 | *Delgado* | *Pepitilla* | *-* | 20 | Tetecala |
| MORE103 | 2034 | *Ancho* | *Vandeño* | *Pepitilla* | 21 | Coatlán del Río |
| MORE104 | 2035 | *Ancho* | *Pepitilla* | *Ancho* | 18 | Coatlán del Río |
| MORE105 | 2036 | *Tehuacán* | *Vandeño* | *-* | 20 | Coatlán del Río |
| MORE106 | 2044 | *Ancho* | *Vandeño* | *-* | 20 | Puente de Ixtla |
| MORE107 | 2045 | *Ancho* | *Vandeño* | *Pepitilla* | 20 | Puente de Ixtla |
| MORE108 | 2046 | *Delgado* | *Pepitilla* | *-* | 20 | Puente de Ixtla |
| MORE109 | 2047 | *Grueso negro* | *Elotes Occidentales* | *Olotillo* | 20 | Puente de Ixtla |
| MORE110 | 2048 | *Grueso colorado* | *Elotes Occidentales* | *Olotillo* | 20 | Puente de Ixtla |
| MORE111 | 2049 | *Grueso blanco* | *Vandeño* | *-* | 20 | Puente de Ixtla |
| MORE112 | 2050 | *Ancho* | *Vandeño* | *Olotillo* | 21 | Amacuzac |
| MORE113 | 2051 | *Delgado* | *Pepitilla* | *-* | 20 | Amacuzac |
| MORE114 | 2052 | *Ancho* | *Ancho* | *Olotillo* | 24 | Amacuzac |
| MORE115 | 2053 | *Ancho* | *Ancho* | *Olotillo* | 24 | Amacuzac |

Based on samples’ passport data from the CIMMYT Germplasm Bank database (http://mgb.cimmyt.org/gringlobal), (Kato, 1967) and farmers’ interviews.

^a^ Race classification by Dr. Rafael Ortega-Pazcka in 2013, based on photographs of each sample. Numbers 5- 9 refer to the degree purity (lack of influence from other established races), from lowest to highest.

* bold lettering; samples included in this study.

# Supplementary Information 2. Comparison of the 13 ex situ samples included in this study with other samples from the 1967 collection using the SeeD genotypes.

Based on an independent dataset of 64 477 SNPs. Genotypes for 91 of the 93 samples in the collection were provided by the Seeds of Discovery (SeeD) initiative (Pixley *et al*. 2018). In the 3^rd^ to 5^th^ columns, private alleles are subdivided based on their MAF (Minor Allele Frequency). Samples found *in situ* in 2017 roughly contain 83.1% of the alleles that were originally present in the collection. Most of the alleles that are absent from the subset that was not found *in situ* in 2017 (78.4%). are minor alleles of low or rare frequency (<=5%).

# Supplementary Information 3. Seed collection questionnaire for farmers growing in situ samples collected in 2017

1. Collection date (mm/dd/yyyy):
2. Plot location (State, municipality, town, reference, GPS coordinates [latitude, longitude, elevation])
3. Years cultivating seed lots:
4. Number of collected ears:
5. Common name(s):
6. Main use (grain, fresh cob, forage, other):
7. Main destination (consumption, market, both, other):
8. Approximate area currently planted with seed lot (ha):
9. Approximate area planted with seed lot in 1967 (ha):
10. Usual planting date or date interval (mm/dd or mm):
11. Usual flowering date or date interval (mm/dd or mm):
12. Usual harvest date or date interval (mm/dd or mm):
13. Approximate distance from own plot to neighboring maize plots:
14. Maize varieties cultivated in neighboring maize plots:
15. Do flowering periods between own and neighboring maize plots usually coincide?
16. When does seed selection for next season’s planting usually take place?
17. How does seed selection for next season’s planting usually take place?
18. Traits chosen for seed selection
    Ears:
    Cob:
    Kernels:
    Plant:
    Other:

# Supplementary Information 4. SNP density by chromosomes based on 74 739 SNPs .

Vertical bars represent SNP density in 1 Mbp windows, with a yellow to orange to red color range, according to legend. Positions and chromosomes are represented in the x and y-axes, respectively. Black triangles represent the positions of centromeres.

# Supplementary Information 5. Genetic diversity indicators per sample pairs.

| Sample pairs | | Primary race | Number of polymorphic SNPs  in sample^2^ | | | Percentage of polymorphic SNPs in sample^2^ | | |  | | He | | | | | | Ho | | | | |
| --- | --- | --- | --- | --- | --- | --- | --- | --- | --- | --- | --- | --- | --- | --- | --- | --- | --- | --- | --- | --- | --- |
|  |  |  | *Ex situ* sample | *In situ* sample | | *Ex situ* sample | | *In situ* sample | | *P*-value | *Ex situ* sample | | *In situ* sample | | *P*-value | | *Ex situ* sample | | *In situ* sample | | *P*-value |
| Location: Tetela del Volcán | | | | | | | | | | | | | | | | | | | | | |
| M32 | *Chalqueño* | | 19 868 | | 21 160 | 26.5% | 28.2% | | | <0.0000 | 0.070 | 0.071 | | 0.0403 | | 0.053 | | 0.060 | | <0.0000 | |
| M33 | *Chalqueño* | | 21 166 | | 23 390 | 28.2% | 31.2% | | | <0.0000 | 0.071 | 0.074 | | 0.0001 | | 0.057 | | 0.057 | | 0.3113 | |
| M34 | *Elotes Cónicos* | | 21 134 | | 20 871 | 28.3% | 27.8% | | | 0.1317 | 0.071 | 0.071 | | 0.2625 | | 0.057 | | 0.050 | | <0.0000 | |
| M35 | *Cónico* | | 21 463 | | 20 510 | 28.6% | 27.4% | | | <0.0000 | 0.070 | 0.066 | | <0.0000 | | 0.058 | | 0.055 | | <0.0000 | |
| M39 | *Pepitilla* | | 21 218 | | 21 336 | 28.3% | 28.5% | | | 0.5025 | 0.071 | 0.070 | | 0.4030 | | 0.055 | | 0.058 | | <0.0000 | |
| Location: Totolapan | | | | | | | | | | | | | | | | | | | | | |
| M44 | *Ancho* | | 20 575 | | 21 699 | 27.4% | 28.9% | | | <0.0000 | 0.068 | 0.069 | | 0.0609 | | 0.056 | | 0.057 | | 0.0666 | |
| M45 | *Ancho* | | 20 721 | | 21 387 | 27.6% | 28.5% | | | 0.0001 | 0.068 | 0.069 | | 0.1382 | | 0.056 | | 0.057 | | 0.0836 | |
| M46 | *Ancho* | | 22 306 | | 21 932 | 29.8% | 29.3% | | | 0.0346 | 0.071 | 0.070 | | 0.1851 | | 0.059 | | 0.057 | | 0.0926 | |
| M47 | *Ancho* | | 21 697 | | 21 933 | 28.9% | 29.3% | | | 0.1812 | 0.069 | 0.070 | | 0.1421 | | 0.056 | | 0.055 | | 0.0470 | |
| M49 | *Ancho* | | 21 572 | | 21 579 | 28.8% | 28.8% | | | 0.9727 | 0.069 | 0.069 | | 0.4296 | | 0.057 | | 0.058 | | 0.0159 | |
| M50 | *Ancho* | | 20 846 | | 22 224 | 27.8% | 29.6% | | | <0.0000 | 0.069 | 0.070 | | 0.0366 | | 0.056 | | 0.058 | | 0.0003 | |
| Location: Tepoztlán | | | | | | | | | | | | | | | | | | | | | |
| M75 | *Ancho* | | 20 896 | | 21 662 | 27.9% | 28.9% | | | <0.0000 | 0.070 | 0.069 | | 0.2711 | | 0.056 | | 0.058 | | 0.0453 | |
| Location: Xochitepec | | | | | | | | | | | | | | | | | | | | | |
| M87 | *Ancho* | | 21 971 | | 18 742 | 29.3% | 25.0% | | | <0.0000 | 0.072 | 0.064 | | <0.0000 | | 0.056 | | 0.052 | | <0.0000 | |

^1^ Each sample combines the genotypes of 10 individual seedlings.

^2^ From a total of 74 739 SNPs in the complete dataset, after monomorphic SNPs for each sample were removed.

# Supplementary Information 6. Site-frequency spectrum of ex situ and in situ samples.

Comparison based on a total of 74 739 SNPs in the complete dataset, after monomorphic SNPs for each subgroup were removed. *P*-value < 2.2e-16 based on a two-sided Kolmogorov-Smirnov test.

# Supplementary Information 7. Alternative color scheme for Figures 2a and 2b.

(a) (b)

(a) Fst-based neighbor joining phylogeny representing the relationship between *ex situ* and *in situ* samples. For each sample, five random seedlings are represented. (b) Principal Component Analysis (PCA) including all samples. PC1 and PC2 are shown on the x and y-axes, respectively, with the percentage of explained variance. Colors and shapes represent each sample’s conservation strategy (*ex situ* or *in situ*) and its race, respectively, according to the legend.

# Supplementary Information 8. Analysis of Molecular Variance (AMOVA).

| **Source of variance** | **Samples** | | **Pairs** | | ***Ex situ* or *in situ* conservation strategy** | | **Race** | | **Municipality** | |
| --- | --- | --- | --- | --- | --- | --- | --- | --- | --- | --- |
|  | σ | % | σ | % | σ | % | σ | % | σ | % |
| Between subgroups | 205.0 | 5.7 | 114.3 | 3.2 | 24.8 | 0.7 | 102.1 | 2.8 | 70.9 | 2.0 |
| Between seedlings within subgroups | 772.5 | 21.6 | 864.5 | 24.2 | 957.9 | 26.8 | 911.0 | 25.3 | 925.7 | 25.8 |
| Within seedlings^1^ | 2 594.7 | 72.6 | 2 594.7 | 72.6 | 2 594.7 | 72.5 | 2 594.7 | 71.9 | 2 594.7 | 72.2 |
| Total | 3 572.2 | 100.0 | 3 573.4 | 100.0 | 3 577.4 | 100.0 | 3 607.7 | 100.0 | 3 591.3 | 100.0 |

^1^ Heterozygosity within individual seedlings (genotypes) was taken into account as a source of variance.

# Supplementary Information 9. Pairwise F_st_ between samples (95% CI).

|  | M32-Ex | | | | M33-Ex | | | | M34-Ex | | | | M35-Ex | | | | M39-Ex | | | | M44-Ex | | | | M45-Ex | | | |
| --- | --- | --- | --- | --- | --- | --- | --- | --- | --- | --- | --- | --- | --- | --- | --- | --- | --- | --- | --- | --- | --- | --- | --- | --- | --- | --- | --- | --- |
| M33-Ex | 0.078 | (0.074 | - | 0.082) |  |  |  |  |  |  |  |  |  |  |  |  |  |  |  |  |  |  |  |  |  |  |  |  |
| M34-Ex | 0.067 | (0.064 | - | 0.071) | **0.049** | **(0.046** | **-** | **0.052)** |  |  |  |  |  |  |  |  |  |  |  |  |  |  |  |  |  |  |  |  |
| M35-Ex | 0.101 | (0.096 | - | 0.107) | 0.068 | (0.064 | - | 0.072) | 0.068 | (0.064 | - | 0.072) |  |  |  |  |  |  |  |  |  |  |  |  |  |  |  |  |
| M39-Ex | 0.099 | (0.094 | - | 0.104) | 0.068 | (0.065 | - | 0.072) | 0.067 | (0.065 | - | 0.070) | 0.052 | (0.049 | - | 0.055) |  |  |  |  |  |  |  |  |  |  |  |  |
| M44-Ex | 0.118 | (0.113 | - | 0.123) | 0.077 | (0.073 | - | 0.081) | 0.084 | (0.080 | - | 0.088) | 0.052 | (0.049 | - | 0.056) | 0.057 | (0.054 | - | 0.061) |  |  |  |  |  |  |  |  |
| M45-Ex | 0.116 | (0.110 | - | 0.122) | 0.078 | (0.073 | - | 0.082) | 0.081 | (0.076 | - | 0.085) | **0.047** | **(0.044** | **-** | **0.050)** | 0.055 | (0.052 | - | 0.058) | **0.044** | **(0.042** | **-** | **0.047)** |  |  |  |  |
| M46-Ex | 0.106 | (0.101 | - | 0.112) | 0.071 | (0.067 | - | 0.075) | 0.075 | (0.071 | - | 0.079) | **0.040** | **(0.037** | **-** | **0.043)** | **0.048** | **(0.045** | **-** | **0.052)** | **0.038** | **(0.036** | **-** | **0.041)** | **0.033** | **(0.030** | **-** | **0.036)** |
| M47-Ex | 0.110 | (0.105 | - | 0.116) | 0.073 | (0.068 | - | 0.077) | 0.076 | (0.072 | - | 0.080) | **0.040** | **(0.037** | **-** | **0.044)** | **0.049** | **(0.046** | **-** | **0.052)** | **0.037** | **(0.035** | **-** | **0.040)** | **0.034** | **(0.031** | **-** | **0.037)** |
| M49-Ex | 0.103 | (0.098 | - | 0.109) | 0.068 | (0.065 | - | 0.072) | 0.071 | (0.067 | - | 0.074) | **0.049** | **(0.046** | **-** | **0.053)** | 0.052 | (0.050 | - | 0.056) | 0.050 | (0.047 | - | 0.053) | **0.048** | **(0.045** | **-** | **0.052)** |
| M50-Ex | 0.108 | (0.103 | - | 0.113) | 0.067 | (0.064 | - | 0.072) | 0.076 | (0.072 | - | 0.081) | 0.060 | (0.056 | - | 0.063) | 0.067 | (0.063 | - | 0.071) | 0.056 | (0.054 | - | 0.059) | 0.056 | (0.052 | - | 0.059) |
| M75-Ex | 0.116 | (0.111 | - | 0.122) | 0.074 | (0.070 | - | 0.078) | 0.081 | (0.077 | - | 0.085) | **0.042** | **(0.039** | **-** | **0.046)** | 0.054 | (0.052 | - | 0.057) | **0.044** | **(0.041** | **-** | **0.047)** | **0.042** | **(0.040** | **-** | **0.044)** |
| M87-Ex | 0.128 | (0.123 | - | 0.134) | 0.079 | (0.074 | - | 0.084) | 0.091 | (0.086 | - | 0.096) | **0.049** | **(0.046** | **-** | **0.053)** | 0.058 | (0.055 | - | 0.062) | **0.047** | **(0.043** | **-** | **0.050)** | **0.042** | **(0.039** | **-** | **0.045)** |
| M32-In | 0.085 | (0.080 | - | 0.089) | 0.063 | (0.059 | - | 0.066) | 0.066 | (0.063 | - | 0.069) | 0.065 | (0.062 | - | 0.069) | 0.070 | (0.067 | - | 0.073) | 0.074 | (0.070 | - | 0.079) | 0.075 | (0.070 | - | 0.080) |
| M33-In | 0.081 | (0.076 | - | 0.086) | **0.046** | **(0.043** | **-** | **0.049)** | 0.053 | (0.050 | - | 0.056) | **0.033** | **(0.029** | **-** | **0.036)** | **0.039** | **(0.036** | **-** | **0.042)** | **0.036** | **(0.034** | **-** | **0.039)** | **0.035** | **(0.032** | **-** | **0.038)** |
| M34-In | 0.085 | (0.081 | - | 0.091) | 0.057 | (0.054 | - | 0.061) | 0.057 | (0.054 | - | 0.060) | 0.053 | (0.049 | - | 0.057) | 0.056 | (0.054 | - | 0.060) | 0.062 | (0.059 | - | 0.065) | 0.058 | (0.056 | - | 0.062) |
| M35-In | 0.145 | (0.140 | - | 0.152) | 0.103 | (0.098 | - | 0.107) | 0.109 | (0.104 | - | 0.113) | 0.067 | (0.064 | - | 0.07) | 0.076 | (0.072 | - | 0.080) | 0.068 | (0.064 | - | 0.070) | 0.059 | (0.056 | - | 0.063) |
| M39-In | 0.102 | (0.097 | - | 0.106) | 0.067 | (0.064 | - | 0.071) | 0.071 | (0.068 | - | 0.074) | **0.046** | **(0.043** | **-** | **0.049)** | 0.057 | (0.054 | - | 0.060) | 0.054 | (0.051 | - | 0.057) | 0.052 | (0.050 | - | 0.055) |
| M44-In | 0.119 | (0.112 | - | 0.124) | 0.078 | (0.074 | - | 0.082) | 0.083 | (0.078 | - | 0.087) | **0.042** | **(0.039** | **-** | **0.045)** | 0.050 | (0.047 | - | 0.053) | **0.046** | **(0.044** | **-** | **0.049)** | **0.042** | **(0.039** | **-** | **0.045)** |
| M45-In | 0.120 | (0.115 | - | 0.126) | 0.078 | (0.075 | - | 0.082) | 0.087 | (0.084 | - | 0.091) | **0.047** | **(0.044** | **-** | **0.050)** | 0.057 | (0.054 | - | 0.060) | 0.050 | (0.047 | - | 0.053) | **0.045** | **(0.042** | **-** | **0.049)** |
| M46-In | 0.109 | (0.104 | - | 0.114) | 0.069 | (0.065 | - | 0.073) | 0.074 | (0.070 | - | 0.078) | **0.036** | **(0.033** | **-** | **0.039)** | **0.045** | **(0.043** | **-** | **0.049)** | **0.035** | **(0.032** | **-** | **0.038)** | **0.032** | **(0.030** | **-** | **0.036)** |
| M47-In | 0.109 | (0.104 | - | 0.115) | 0.067 | (0.063 | - | 0.071) | 0.074 | (0.069 | - | 0.077) | **0.039** | **(0.036** | **-** | **0.042)** | **0.049** | **(0.046** | **-** | **0.053)** | **0.038** | **(0.035** | **-** | **0.041)** | **0.034** | **(0.032** | **-** | **0.036)** |
| M49-In | 0.109 | (0.105 | - | 0.114) | 0.073 | (0.070 | - | 0.077) | 0.081 | (0.077 | - | 0.084) | **0.046** | **(0.043** | **-** | **0.049)** | 0.054 | (0.051 | - | 0.057) | **0.043** | **(0.040** | **-** | **0.047)** | **0.039** | **(0.037** | **-** | **0.042)** |
| M50-In | 0.104 | (0.099 | - | 0.109) | 0.067 | (0.064 | - | 0.071) | 0.072 | (0.068 | - | 0.076) | **0.035** | **(0.032** | **-** | **0.039)** | **0.043** | **(0.041** | **-** | **0.046)** | **0.035** | **(0.032** | **-** | **0.038)** | **0.030** | **(0.028** | **-** | **0.033)** |
| M75-In | 0.123 | (0.118 | - | 0.129) | 0.079 | (0.074 | - | 0.083) | 0.089 | (0.085 | - | 0.092) | **0.049** | **(0.045** | **-** | **0.052)** | 0.059 | (0.056 | - | 0.062) | **0.047** | **(0.044** | **-** | **0.050)** | **0.044** | **(0.041** | **-** | **0.046)** |
| M87-In | 0.164 | (0.158 | - | 0.170) | 0.117 | (0.113 | - | 0.122) | 0.121 | (0.115 | - | 0.125) | 0.080 | (0.077 | - | 0.085) | 0.094 | (0.089 | - | 0.098) | 0.079 | (0.076 | - | 0.083) | 0.079 | (0.076 | - | 0.083) |

Bold = little genetic differentiation (F_st_ < 0.05); regular = moderate genetic differentiation (F_st_ = 0.05 – 0.15); gray background = great genetic differentiation (F_st_ > 0.15), based on Hartl and Clark (1997). Underlined = Comparison between *ex situ* and *in situ* sample pairs.

|  | M46-Ex | | | | M47-Ex | | | | M49-Ex | | | | M50-Ex | | | | M75-Ex | | | | M87-Ex | | | | M32-In | | | |
| --- | --- | --- | --- | --- | --- | --- | --- | --- | --- | --- | --- | --- | --- | --- | --- | --- | --- | --- | --- | --- | --- | --- | --- | --- | --- | --- | --- | --- |
| M33-Ex |  |  |  |  |  |  |  |  |  |  |  |  |  |  |  |  |  |  |  |  |  |  |  |  |  |  |  |  |
| M34-Ex |  |  |  |  |  |  |  |  |  |  |  |  |  |  |  |  |  |  |  |  |  |  |  |  |  |  |  |  |
| M35-Ex |  |  |  |  |  |  |  |  |  |  |  |  |  |  |  |  |  |  |  |  |  |  |  |  |  |  |  |  |
| M39-Ex |  |  |  |  |  |  |  |  |  |  |  |  |  |  |  |  |  |  |  |  |  |  |  |  |  |  |  |  |
| M44-Ex |  |  |  |  |  |  |  |  |  |  |  |  |  |  |  |  |  |  |  |  |  |  |  |  |  |  |  |  |
| M45-Ex |  |  |  |  |  |  |  |  |  |  |  |  |  |  |  |  |  |  |  |  |  |  |  |  |  |  |  |  |
| M46-Ex |  |  |  |  |  |  |  |  |  |  |  |  |  |  |  |  |  |  |  |  |  |  |  |  |  |  |  |  |
| M47-Ex | **0.000** | **(-0.004** | **-** | **0.000)** |  |  |  |  |  |  |  |  |  |  |  |  |  |  |  |  |  |  |  |  |  |  |  |  |
| M49-Ex | **0.042** | **(0.039** | **-** | **0.045)** | **0.043** | **(0.039** | **-** | **0.046)** |  |  |  |  |  |  |  |  |  |  |  |  |  |  |  |  |  |  |  |  |
| M50-Ex | **0.047** | **(0.044** | **-** | **0.050)** | 0.052 | (0.050 | - | 0.055) | 0.051 | (0.048 | - | 0.055) |  |  |  |  |  |  |  |  |  |  |  |  |  |  |  |  |
| M75-Ex | **0.031** | **(0.029** | **-** | **0.034)** | **0.034** | **(0.031** | **-** | **0.037)** | **0.041** | **(0.038** | **-** | **0.044)** | **0.048** | **(0.044** | **-** | **0.051)** |  |  |  |  |  |  |  |  |  |  |  |  |
| M87-Ex | **0.034** | **(0.030** | **-** | **0.036)** | **0.041** | **(0.037** | **-** | **0.044)** | 0.050 | (0.047 | - | 0.054) | 0.061 | (0.058 | - | 0.065) | **0.040** | **(0.036** | **-** | **0.043)** |  |  |  |  |  |  |  |  |
| M32-In | 0.067 | (0.064 | - | 0.071) | 0.071 | (0.067 | - | 0.075) | 0.072 | (0.069 | - | 0.076) | 0.069 | (0.066 | - | 0.073) | 0.075 | (0.071 | - | 0.079) | 0.081 | (0.076 | - | 0.086) |  |  |  |  |
| M33-In | **0.023** | **(0.021** | **-** | **0.026)** | **0.032** | **(0.028** | **-** | **0.034)** | **0.033** | **(0.031** | **-** | **0.036)** | **0.039** | **(0.037** | **-** | **0.042)** | **0.031** | **(0.029** | **-** | **0.034)** | **0.034** | **(0.031** | **-** | **0.037)** | **0.048** | **(0.044** | **-** | **0.051)** |
| M34-In | 0.052 | (0.050 | - | 0.056) | 0.053 | (0.051 | - | 0.057) | 0.056 | (0.053 | - | 0.060) | 0.060 | (0.055 | - | 0.063) | 0.057 | (0.054 | - | 0.061) | 0.062 | (0.059 | - | 0.066) | 0.058 | (0.055 | - | 0.062) |
| M35-In | 0.052 | (0.049 | - | 0.055) | 0.059 | (0.055 | - | 0.062) | 0.071 | (0.068 | - | 0.074) | 0.081 | (0.077 | - | 0.084) | 0.062 | (0.060 | - | 0.065) | 0.053 | (0.049 | - | 0.056) | 0.101 | (0.096 | - | 0.105) |
| M39-In | **0.041** | **(0.038** | **-** | **0.044)** | **0.045** | **(0.042** | **-** | **0.049)** | 0.055 | (0.051 | - | 0.059) | 0.062 | (0.058 | - | 0.066) | 0.053 | (0.049 | - | 0.055) | 0.052 | (0.048 | - | 0.055) | 0.064 | (0.061 | - | 0.067) |
| M44-In | **0.028** | **(0.025** | **-** | **0.031)** | **0.032** | **(0.029** | **-** | **0.035)** | **0.047** | **(0.044** | **-** | **0.050)** | 0.055 | (0.051 | - | 0.059) | **0.037** | **(0.034** | **-** | **0.040)** | **0.043** | **(0.040** | **-** | **0.047)** | 0.072 | (0.068 | - | 0.076) |
| M45-In | **0.030** | **(0.027** | **-** | **0.033)** | **0.035** | **(0.033** | **-** | **0.038)** | 0.051 | (0.048 | - | 0.054) | 0.055 | (0.052 | - | 0.058) | **0.037** | **(0.034** | **-** | **0.040)** | **0.041** | **(0.038** | **-** | **0.045)** | 0.074 | (0.070 | - | 0.078) |
| M46-In | **0.021** | **(0.019** | **-** | **0.024)** | **0.028** | **(0.026** | **-** | **0.032)** | **0.037** | **(0.034** | **-** | **0.040)** | **0.046** | **(0.042** | **-** | **0.049)** | **0.025** | **(0.023** | **-** | **0.027)** | **0.029** | **(0.025** | **-** | **0.032)** | 0.065 | (0.062 | - | 0.069) |
| M47-In | **0.025** | **(0.023** | **-** | **0.028)** | **0.030** | **(0.027** | **-** | **0.033)** | **0.039** | **(0.036** | **-** | **0.042)** | **0.042** | **(0.038** | **-** | **0.044)** | **0.029** | **(0.026** | **-** | **0.031)** | **0.034** | **(0.031** | **-** | **0.036)** | 0.063 | (0.060 | - | 0.067) |
| M49-In | **0.030** | **(0.027** | **-** | **0.033)** | **0.037** | **(0.034** | **-** | **0.040)** | **0.045** | **(0.042** | **-** | **0.048)** | **0.055** | **(0.052** | **-** | **0.058)** | **0.036** | **(0.033** | **-** | **0.038)** | **0.042** | **(0.037** | **-** | **0.045)** | 0.067 | (0.063 | - | 0.071) |
| M50-In | **0.018** | **(0.016** | **-** | **0.021)** | **0.025** | **(0.022** | **-** | **0.028)** | **0.036** | **(0.034** | **-** | **0.039)** | **0.041** | **(0.038** | **-** | **0.044)** | **0.023** | **(0.021** | **-** | **0.026)** | **0.028** | **(0.025** | **-** | **0.031)** | 0.063 | (0.060 | - | 0.067) |
| M75-In | **0.034** | **(0.031** | **-** | **0.037)** | **0.042** | **(0.038** | **-** | **0.045)** | **0.049** | **(0.046** | **-** | **0.052)** | 0.058 | (0.054 | - | 0.062) | **0.033** | **(0.030** | **-** | **0.036)** | **0.040** | **(0.036** | **-** | **0.042)** | 0.077 | (0.073 | - | 0.081) |
| M87-In | 0.063 | (0.059 | - | 0.066) | 0.070 | (0.066 | - | 0.074) | 0.089 | (0.086 | - | 0.094) | 0.097 | (0.091 | - | 0.101) | 0.081 | (0.077 | - | 0.085) | 0.064 | (0.060 | - | 0.067) | 0.113 | (0.109 | - | 0.117) |

Bold = little genetic differentiation (F_st_ < 0.05); regular = moderate genetic differentiation (F_st_ = 0.05 – 0.15); gray background = great genetic differentiation (F_st_ > 0.15), based on Hartl and Clark (1997). Underlined = Comparison between *ex situ* and *in situ* sample pairs.

|  | M33-In | | | | M34-In | | | | M35-In | | | | M39-In | | | | M44-In | | | | M45-In | | | | M46-In | | | |
| --- | --- | --- | --- | --- | --- | --- | --- | --- | --- | --- | --- | --- | --- | --- | --- | --- | --- | --- | --- | --- | --- | --- | --- | --- | --- | --- | --- | --- |
| M33-Ex |  |  |  |  |  |  |  |  |  |  |  |  |  |  |  |  |  |  |  |  |  |  |  |  |  |  |  |  |
| M34-Ex |  |  |  |  |  |  |  |  |  |  |  |  |  |  |  |  |  |  |  |  |  |  |  |  |  |  |  |  |
| M35-Ex |  |  |  |  |  |  |  |  |  |  |  |  |  |  |  |  |  |  |  |  |  |  |  |  |  |  |  |  |
| M39-Ex |  |  |  |  |  |  |  |  |  |  |  |  |  |  |  |  |  |  |  |  |  |  |  |  |  |  |  |  |
| M44-Ex |  |  |  |  |  |  |  |  |  |  |  |  |  |  |  |  |  |  |  |  |  |  |  |  |  |  |  |  |
| M45-Ex |  |  |  |  |  |  |  |  |  |  |  |  |  |  |  |  |  |  |  |  |  |  |  |  |  |  |  |  |
| M46-Ex |  |  |  |  |  |  |  |  |  |  |  |  |  |  |  |  |  |  |  |  |  |  |  |  |  |  |  |  |
| M47-Ex |  |  |  |  |  |  |  |  |  |  |  |  |  |  |  |  |  |  |  |  |  |  |  |  |  |  |  |  |
| M49-Ex |  |  |  |  |  |  |  |  |  |  |  |  |  |  |  |  |  |  |  |  |  |  |  |  |  |  |  |  |
| M50-Ex |  |  |  |  |  |  |  |  |  |  |  |  |  |  |  |  |  |  |  |  |  |  |  |  |  |  |  |  |
| M75-Ex |  |  |  |  |  |  |  |  |  |  |  |  |  |  |  |  |  |  |  |  |  |  |  |  |  |  |  |  |
| M87-Ex |  |  |  |  |  |  |  |  |  |  |  |  |  |  |  |  |  |  |  |  |  |  |  |  |  |  |  |  |
| M32-In |  |  |  |  |  |  |  |  |  |  |  |  |  |  |  |  |  |  |  |  |  |  |  |  |  |  |  |  |
| M33-In |  |  |  |  |  |  |  |  |  |  |  |  |  |  |  |  |  |  |  |  |  |  |  |  |  |  |  |  |
| M34-In | **0.030** | **(0.026** | **-** | **0.032)** |  |  |  |  |  |  |  |  |  |  |  |  |  |  |  |  |  |  |  |  |  |  |  |  |
| M35-In | 0.053 | (0.051 | - | 0.056) | 0.082 | (0.078 | - | 0.086) |  |  |  |  |  |  |  |  |  |  |  |  |  |  |  |  |  |  |  |  |
| M39-In | **0.031** | **(0.029** | **-** | **0.034)** | 0.054 | (0.051 | - | 0.058) | 0.071 | (0.067 | - | 0.073) |  |  |  |  |  |  |  |  |  |  |  |  |  |  |  |  |
| M44-In | **0.025** | **(0.022** | **-** | **0.028)** | 0.055 | (0.053 | - | 0.059) | 0.061 | (0.058 | - | 0.065) | **0.049** | **(0.046** | **-** | **0.052)** |  |  |  |  |  |  |  |  |  |  |  |  |
| M45-In | **0.025** | **(0.023** | **-** | **0.027)** | 0.058 | (0.055 | - | 0.061) | 0.068 | (0.065 | - | 0.071) | **0.047** | **(0.043** | **-** | **0.050)** | **0.016** | **(0.013** | **-** | **0.019)** |  |  |  |  |  |  |  |  |
| M46-In | **0.022** | **(0.019** | **-** | **0.025)** | **0.049** | **(0.046** | **-** | **0.053)** | 0.054 | (0.051 | - | 0.057) | **0.042** | **(0.039** | **-** | **0.045)** | **0.018** | **(0.016** | **-** | **0.021)** | **0.022** | **(0.020** | **-** | **0.025)** |  |  |  |  |
| M47-In | **0.020** | **(0.017** | **-** | **0.022)** | **0.049** | **(0.046** | **-** | **0.052)** | 0.054 | (0.051 | - | 0.057) | **0.037** | **(0.035** | **-** | **0.040)** | **0.019** | **(0.016** | **-** | **0.022)** | **0.023** | **(0.020** | **-** | **0.026)** | **0.018** | **(0.016** | **-** | **0.021)** |
| M49-In | **0.026** | **(0.024** | **-** | **0.029)** | 0.053 | (0.050 | - | 0.057) | 0.064 | (0.061 | - | 0.067) | **0.045** | **(0.042** | **-** | **0.048)** | **0.022** | **(0.019** | **-** | **0.024)** | **0.027** | **(0.025** | **-** | **0.030)** | **0.025** | **(0.022** | **-** | **0.028)** |
| M50-In | **0.017** | **(0.014** | **-** | **0.019)** | **0.046** | **(0.043** | **-** | **0.050)** | 0.053 | (0.050 | - | 0.056) | **0.038** | **(0.035** | **-** | **0.040)** | **0.015** | **(0.012** | **-** | **0.017)** | **0.018** | **(0.016** | **-** | **0.021)** | **0.008** | **(0.006** | **-** | **0.010)** |
| M75-In | **0.032** | **(0.029** | **-** | **0.035)** | 0.066 | (0.063 | - | 0.070) | **0.043** | **(0.040** | **-** | **0.045)** | 0.052 | (0.049 | - | 0.055) | **0.044** | **(0.041** | **-** | **0.047)** | **0.043** | **(0.041** | **-** | **0.046)** | **0.027** | **(0.025** | **-** | **0.030)** |
| M87-In | 0.069 | (0.066 | - | 0.073) | 0.099 | (0.095 | - | 0.103) | **0.049** | **(0.046** | **-** | **0.053)** | 0.086 | (0.081 | - | 0.090) | 0.079 | (0.075 | - | 0.083) | 0.080 | (0.076 | - | 0.083) | 0.064 | (0.061 | - | 0.069) |

Bold = little genetic differentiation (F_st_ < 0.05); regular = moderate genetic differentiation (F_st_ = 0.05 – 0.15); gray background = great genetic differentiation (F_st_ > 0.15), based on Hartl and Clark (1997). Underlined = Comparison between *ex situ* and *in situ* sample pairs.

|  | M47-In | | | | M49-In | | | | M50-In | | | | M75-In | | | |
| --- | --- | --- | --- | --- | --- | --- | --- | --- | --- | --- | --- | --- | --- | --- | --- | --- |
| M33-Ex |  |  |  |  |  |  |  |  |  |  |  |  |  |  |  |  |
| M34-Ex |  |  |  |  |  |  |  |  |  |  |  |  |  |  |  |  |
| M35-Ex |  |  |  |  |  |  |  |  |  |  |  |  |  |  |  |  |
| M39-Ex |  |  |  |  |  |  |  |  |  |  |  |  |  |  |  |  |
| M44-Ex |  |  |  |  |  |  |  |  |  |  |  |  |  |  |  |  |
| M45-Ex |  |  |  |  |  |  |  |  |  |  |  |  |  |  |  |  |
| M46-Ex |  |  |  |  |  |  |  |  |  |  |  |  |  |  |  |  |
| M47-Ex |  |  |  |  |  |  |  |  |  |  |  |  |  |  |  |  |
| M49-Ex |  |  |  |  |  |  |  |  |  |  |  |  |  |  |  |  |
| M50-Ex |  |  |  |  |  |  |  |  |  |  |  |  |  |  |  |  |
| M75-Ex |  |  |  |  |  |  |  |  |  |  |  |  |  |  |  |  |
| M87-Ex |  |  |  |  |  |  |  |  |  |  |  |  |  |  |  |  |
| M32-In |  |  |  |  |  |  |  |  |  |  |  |  |  |  |  |  |
| M33-In |  |  |  |  |  |  |  |  |  |  |  |  |  |  |  |  |
| M34-In |  |  |  |  |  |  |  |  |  |  |  |  |  |  |  |  |
| M35-In |  |  |  |  |  |  |  |  |  |  |  |  |  |  |  |  |
| M39-In |  |  |  |  |  |  |  |  |  |  |  |  |  |  |  |  |
| M44-In |  |  |  |  |  |  |  |  |  |  |  |  |  |  |  |  |
| M45-In |  |  |  |  |  |  |  |  |  |  |  |  |  |  |  |  |
| M46-In |  |  |  |  |  |  |  |  |  |  |  |  |  |  |  |  |
| M47-In |  |  |  |  |  |  |  |  |  |  |  |  |  |  |  |  |
| M49-In | **0.024** | **(0.021** | **-** | **0.027)** |  |  |  |  |  |  |  |  |  |  |  |  |
| M50-In | **0.015** | **(0.013** | **-** | **0.017)** | **0.014** | **(0.012** | **-** | **0.016)** |  |  |  |  |  |  |  |  |
| M75-In | **0.035** | **(0.032** | **-** | **0.038)** | **0.045** | **(0.042** | **-** | **0.049)** | **0.027** | **(0.024** | **-** | **0.030)** |  |  |  |  |
| M87-In | 0.071 | (0.068 | - | 0.075) | 0.081 | (0.078 | - | 0.086) | 0.064 | (0.060 | - | 0.067) | 0.056 | (0.053 | - | 0.059) |

Bold = little genetic differentiation (F_st_ < 0.05); regular = moderate genetic differentiation (F_st_ = 0.05 – 0.15); gray background = great genetic differentiation (F_st_ > 0.15), based on Hartl and Clark (1997). Underlined = Comparison between *ex situ* and *in situ* sample pairs.

# Supplementary Information 10. F_st_-based neighbor joining phylogeny.

F_st_-based neighbor joining phylogeny representing the relationship between *ex situ* and *in situ* samples. Colors and shapes represent each sample’s conservation strategy (*ex situ* or *in situ*) and its race, respectively, according to the legend.

# Supplementary Information 11. Bayesian Information Criterion from the DAPC.

Minimum value indicates the optimum number of clusters to retain in the DAPC.

# Supplementary Information 12. Rates of linkage disequilibrium (LD) decay per chromosome within the set.

| Chromosome | Maximum LD (*r^2^*) | LD decay distance (Kb, *r^2^* = 0.1) |
| --- | --- | --- |
| 1 | 0.4571 | 5.5 |
| 2 | 0.4571 | 6.5 |
| 3 | 0.4571 | 5.9 |
| 4 | 0.4571 | 5.9 |
| 5 | 0.4569 | 4.7 |
| 6 | 0.4571 | 6.3 |
| 7 | 0.4571 | 6.9 |
| 8 | 0.4570 | 4.1 |
| 9 | 0.4573 | 13.2 |
| 10 | 0.4569 | 3.6 |

# Supplementary Information 13. Favorable and unfavorable characteristics associated to the three landraces with the most distinct phenotypes from the 1967 collection.

| **Characteristics** |  | ***Ancho* (n=19)** | ***Pepitilla* (n=19)** | ***Negro* (n=18)** |
| --- | --- | --- | --- | --- |
| **Favorable** |  |  |  |  |
| Kernel sweetness | High | 58% | 42% | 100% |
| Market price | High | 37% |  | 11% |
| Use in special preparations | Yes | 26% |  | 28% |
| Kernel softness | High | 21% | 16% | 17% |
| Kernel length and width^a^ | High | 21% | 5% |  |
| Volume yield | High | 11% | 74% |  |
| Ear health^a^ | High | 11% | 11% | 6% |
| Resistance to corn smut | Low | 11% | 5% | 6% |
| Ear length and width^a^ | High | 5% | 11% |  |
| Resistance to pests | High | 5% | 5% | 6% |
| Cob width^a^ | Low |  | 58% |  |
| Ease to shell | High |  | 37% |  |
| Adaptation to local environment | High |  | 11% | 6% |
| Kernel width | Low |  | 11% |  |
| Color^a^ | White (*Ancho*, *Pepitilla*), black (*Negro*) |  | 5% | 6% |
| Kernel set^a^ | Complete |  | 5% |  |
| Uncooked to nixtamalized yield | High |  | 5% |  |
| Market demand | High |  |  | 11% |
| Days as fresh cob | High |  |  | 6% |
| Unfavorable |  |  |  |  |
| Susceptibility to stalk lodging | High | 53% | 53% | 28% |
| Resistance to grain pest | Low | 37% | 37% | 22% |
| Weight yield | Low | 26% | 32% | 28% |
| Resistance to drought | Low | 16% | 16% | 33% |
| Grain to dough yield | Low | 16% | 16% |  |
| Susceptibility to being stolen | High | 16% | 5% | 22% |
| Adaptation to local environment | Low | 16% | 5% | 11% |
| Adaptation to high input system | Low | 11% | 5% | 6% |
| Uncooked to nixtamalized yield | Low | 11% | 5% |  |
| Husk coverage | Poor | 5% |  | 6% |
| Space between kernels | High | 5% |  |  |
| Ease to shell | High | 5% |  |  |
| Kernel length and shape^a^ | High / spiky |  | 47% |  |
| Resistance to high humidity | Low |  | 5% | 11% |
| Market price | Low |  | 5% |  |
| Days as fresh cob | Low |  | 5% |  |
| Rejection in public mills because of color | High |  |  | 33% |
| Ear length and width | Low |  |  | 11% |
| Resistance to frost | Low |  |  | 6% |
| Market demand | Low |  |  | 6% |
| Number of female inflorescences | High |  |  | 6% |

Based on focus group discussion. Numbers of focus groups where each trait was mentioned are expressed as a percentage of the number of focus groups where each landrace had been or was still being cultivated (corresponding column n)

^a^ Traits farmers select for.

# Supplementary Information 14. Loci displaying evidence of selection between ex situ and in situ samples identified with R/OutFLANK.

| **Chr** | **Position** | **He** | **FST** | **q-values** | **p-values** | **p- values Right Tail** |
| --- | --- | --- | --- | --- | --- | --- |
| 1 | 7033020 | 0.2180 | 0.1136 | 0.0093 | 0.0001 | 0.0000 |
| 1 | 15972591 | 0.3034 | 0.0866 | 0.0346 | 0.0005 | 0.0003 |
| 1 | 15973029 | 0.3110 | 0.0803 | 0.0461 | 0.0009 | 0.0004 |
| 1 | 17148269 | 0.1737 | 0.0843 | 0.0346 | 0.0005 | 0.0003 |
| 1 | 19657296 | 0.4646 | 0.0790 | 0.0446 | 0.0008 | 0.0004 |
| 1 | 28059116 | 0.2204 | 0.1046 | 0.0143 | 0.0001 | 0.0001 |
| 1 | 63325826 | 0.1200 | 0.1125 | 0.0088 | 0.0000 | 0.0000 |
| 1 | 72899341 | 0.1356 | 0.1153 | 0.0070 | 0.0000 | 0.0000 |
| 1 | 75647127 | 0.3830 | 0.0930 | 0.0283 | 0.0003 | 0.0002 |
| 1 | 88444223 | 0.2542 | 0.0852 | 0.0390 | 0.0007 | 0.0003 |
| 1 | 88444666 | 0.2550 | 0.0870 | 0.0348 | 0.0006 | 0.0003 |
| 1 | 105549996 | 0.3760 | 0.0888 | 0.0326 | 0.0004 | 0.0002 |
| 1 | 162769006 | 0.1601 | 0.1429 | 0.0018 | 0.0000 | 0.0000 |
| 1 | 162769007 | 0.1594 | 0.1403 | 0.0021 | 0.0000 | 0.0000 |
| 1 | 170172930 | 0.3709 | 0.0994 | 0.0188 | 0.0002 | 0.0001 |
| 1 | 170172947 | 0.3800 | 0.0874 | 0.0326 | 0.0005 | 0.0002 |
| 1 | 170173008 | 0.3678 | 0.1016 | 0.0162 | 0.0001 | 0.0001 |
| 1 | 170173357 | 0.3636 | 0.0983 | 0.0201 | 0.0002 | 0.0001 |
| 1 | 170173374 | 0.3780 | 0.0891 | 0.0317 | 0.0004 | 0.0002 |
| 1 | 170173381 | 0.3699 | 0.0932 | 0.0259 | 0.0003 | 0.0001 |
| 1 | 170173451 | 0.3667 | 0.1025 | 0.0160 | 0.0001 | 0.0001 |
| 1 | 233136455 | 0.4599 | 0.0791 | 0.0490 | 0.0010 | 0.0005 |
| 1 | 242346519 | 0.1070 | 0.0793 | 0.0446 | 0.0008 | 0.0004 |
| 1 | 242346540 | 0.1200 | 0.0945 | 0.0227 | 0.0002 | 0.0001 |
| 1 | 249201121 | 0.3771 | 0.0892 | 0.0318 | 0.0004 | 0.0002 |
| 1 | 252951462 | 0.1635 | 0.0833 | 0.0361 | 0.0006 | 0.0003 |
| 1 | 252951498 | 0.1635 | 0.0833 | 0.0361 | 0.0006 | 0.0003 |
| 2 | 10169794 | 0.1252 | 0.0806 | 0.0446 | 0.0008 | 0.0004 |
| 2 | 28883431 | 0.3212 | 0.0997 | 0.0162 | 0.0001 | 0.0001 |
| 2 | 62408332 | 0.3165 | 0.0848 | 0.0339 | 0.0005 | 0.0002 |
| 2 | 135104516 | 0.1219 | 0.0965 | 0.0203 | 0.0002 | 0.0001 |
| 2 | 135104525 | 0.1219 | 0.0965 | 0.0203 | 0.0002 | 0.0001 |
| 2 | 135104588 | 0.1308 | 0.1086 | 0.0098 | 0.0001 | 0.0000 |
| 2 | 135104596 | 0.1314 | 0.1079 | 0.0102 | 0.0001 | 0.0000 |
| 2 | 188121303 | 0.1069 | 0.0799 | 0.0446 | 0.0008 | 0.0004 |
| 2 | 188163128 | 0.1105 | 0.0941 | 0.0237 | 0.0002 | 0.0001 |
| 2 | 231773241 | 0.1163 | 0.0915 | 0.0290 | 0.0004 | 0.0002 |
| 3 | 8515947 | 0.1601 | 0.1050 | 0.0116 | 0.0001 | 0.0000 |
| 3 | 8515960 | 0.2127 | 0.1147 | 0.0073 | 0.0000 | 0.0000 |
| 3 | 8515970 | 0.1513 | 0.0788 | 0.0446 | 0.0008 | 0.0004 |
| 3 | 8515984 | 0.1241 | 0.0774 | 0.0461 | 0.0009 | 0.0005 |
| 3 | 8516374 | 0.2054 | 0.1456 | 0.0017 | 0.0000 | 0.0000 |
| 3 | 8516376 | 0.1971 | 0.1335 | 0.0026 | 0.0000 | 0.0000 |
| 3 | 8516402 | 0.2096 | 0.1353 | 0.0026 | 0.0000 | 0.0000 |
| 3 | 8516410 | 0.1971 | 0.1463 | 0.0017 | 0.0000 | 0.0000 |
| 3 | 8516435 | 0.1861 | 0.1379 | 0.0024 | 0.0000 | 0.0000 |
| 3 | 20823386 | 0.1430 | 0.0844 | 0.0326 | 0.0005 | 0.0002 |
| 3 | 126667248 | 0.4398 | 0.1233 | 0.0056 | 0.0000 | 0.0000 |
| 3 | 126667320 | 0.3699 | 0.1646 | 0.0011 | 0.0000 | 0.0000 |
| 3 | 126667330 | 0.2999 | 0.1211 | 0.0062 | 0.0000 | 0.0000 |
| 3 | 132757465 | 0.1321 | 0.0911 | 0.0259 | 0.0003 | 0.0001 |
| 3 | 132757814 | 0.1303 | 0.0909 | 0.0259 | 0.0003 | 0.0001 |
| 3 | 135477865 | 0.2881 | 0.1094 | 0.0102 | 0.0001 | 0.0000 |
| 3 | 159170555 | 0.1513 | 0.0826 | 0.0390 | 0.0007 | 0.0003 |
| 3 | 166641116 | 0.4931 | 0.1235 | 0.0055 | 0.0000 | 0.0000 |
| 3 | 166641611 | 0.4993 | 0.1333 | 0.0026 | 0.0000 | 0.0000 |
| 3 | 206550120 | 0.1426 | 0.0829 | 0.0346 | 0.0006 | 0.0003 |
| 3 | 224165059 | 0.4625 | 0.0782 | 0.0461 | 0.0009 | 0.0005 |
| 3 | 229599031 | 0.3687 | 0.1278 | 0.0047 | 0.0000 | 0.0000 |
| 4 | 10605897 | 0.4152 | 0.1356 | 0.0026 | 0.0000 | 0.0000 |
| 4 | 16308861 | 0.2471 | 0.0883 | 0.0318 | 0.0004 | 0.0002 |
| 4 | 23370529 | 0.2449 | 0.0927 | 0.0251 | 0.0003 | 0.0001 |
| 4 | 29282142 | 0.2212 | 0.0918 | 0.0259 | 0.0003 | 0.0001 |
| 4 | 37683701 | 0.1837 | 0.0899 | 0.0283 | 0.0003 | 0.0002 |
| 4 | 100426765 | 0.1443 | 0.1059 | 0.0145 | 0.0001 | 0.0001 |
| 4 | 120555577 | 0.4372 | 0.0962 | 0.0221 | 0.0002 | 0.0001 |
| 4 | 127893287 | 0.2143 | 0.0942 | 0.0259 | 0.0003 | 0.0001 |
| 4 | 127893307 | 0.2143 | 0.0942 | 0.0259 | 0.0003 | 0.0001 |
| 4 | 127893337 | 0.2108 | 0.0900 | 0.0317 | 0.0004 | 0.0002 |
| 4 | 170748561 | 0.4970 | 0.1276 | 0.0048 | 0.0000 | 0.0000 |
| 4 | 174707900 | 0.2673 | 0.0809 | 0.0459 | 0.0009 | 0.0004 |
| 4 | 174856891 | 0.2330 | 0.1400 | 0.0024 | 0.0000 | 0.0000 |
| 4 | 177644199 | 0.4589 | 0.0986 | 0.0227 | 0.0002 | 0.0001 |
| 4 | 177644233 | 0.4495 | 0.1075 | 0.0132 | 0.0001 | 0.0000 |
| 4 | 177644513 | 0.4495 | 0.1075 | 0.0132 | 0.0001 | 0.0000 |
| 4 | 178259503 | 0.3644 | 0.0816 | 0.0425 | 0.0007 | 0.0004 |
| 4 | 180285774 | 0.4337 | 0.1184 | 0.0068 | 0.0000 | 0.0000 |
| 4 | 180285787 | 0.4316 | 0.1143 | 0.0084 | 0.0000 | 0.0000 |
| 4 | 180353205 | 0.4610 | 0.0852 | 0.0346 | 0.0006 | 0.0003 |
| 4 | 180353234 | 0.4643 | 0.0879 | 0.0326 | 0.0004 | 0.0002 |
| 4 | 180355178 | 0.4012 | 0.1089 | 0.0105 | 0.0001 | 0.0000 |
| 4 | 180355219 | 0.4315 | 0.1138 | 0.0084 | 0.0000 | 0.0000 |
| 4 | 184160744 | 0.3024 | 0.0927 | 0.0259 | 0.0003 | 0.0001 |
| 4 | 184365702 | 0.1989 | 0.0883 | 0.0326 | 0.0005 | 0.0002 |
| 4 | 184365732 | 0.1989 | 0.0883 | 0.0326 | 0.0005 | 0.0002 |
| 4 | 184366125 | 0.1989 | 0.0883 | 0.0326 | 0.0005 | 0.0002 |
| 4 | 184366204 | 0.1656 | 0.0964 | 0.0232 | 0.0002 | 0.0001 |
| 4 | 184366205 | 0.1634 | 0.0831 | 0.0422 | 0.0007 | 0.0004 |
| 4 | 184366745 | 0.1634 | 0.0951 | 0.0248 | 0.0002 | 0.0001 |
| 4 | 245085903 | 0.1665 | 0.1068 | 0.0106 | 0.0001 | 0.0000 |
| 5 | 4779712 | 0.1034 | 0.0821 | 0.0361 | 0.0006 | 0.0003 |
| 5 | 6549584 | 0.1210 | 0.0778 | 0.0461 | 0.0009 | 0.0005 |
| 5 | 6549784 | 0.1199 | 0.0769 | 0.0474 | 0.0010 | 0.0005 |
| 5 | 6549840 | 0.1278 | 0.0860 | 0.0326 | 0.0005 | 0.0002 |
| 5 | 17053216 | 0.4008 | 0.0867 | 0.0346 | 0.0005 | 0.0003 |
| 5 | 25432978 | 0.1059 | 0.0779 | 0.0450 | 0.0009 | 0.0004 |
| 5 | 41677640 | 0.4711 | 0.0857 | 0.0346 | 0.0006 | 0.0003 |
| 5 | 41677663 | 0.4702 | 0.0821 | 0.0433 | 0.0008 | 0.0004 |
| 5 | 42792594 | 0.2367 | 0.1961 | 0.0001 | 0.0000 | 0.0000 |
| 5 | 66663523 | 0.2907 | 0.0805 | 0.0463 | 0.0009 | 0.0005 |
| 5 | 131964192 | 0.1333 | 0.1205 | 0.0055 | 0.0000 | 0.0000 |
| 5 | 144546493 | 0.4317 | 0.1334 | 0.0026 | 0.0000 | 0.0000 |
| 5 | 153346518 | 0.1604 | 0.0989 | 0.0160 | 0.0001 | 0.0001 |
| 5 | 153346543 | 0.1604 | 0.0989 | 0.0160 | 0.0001 | 0.0001 |
| 5 | 183397674 | 0.4293 | 0.1365 | 0.0026 | 0.0000 | 0.0000 |
| 5 | 183397702 | 0.4161 | 0.1153 | 0.0081 | 0.0000 | 0.0000 |
| 5 | 184241319 | 0.4196 | 0.0936 | 0.0227 | 0.0002 | 0.0001 |
| 5 | 184241359 | 0.2319 | 0.1471 | 0.0017 | 0.0000 | 0.0000 |
| 5 | 195720678 | 0.2858 | 0.1090 | 0.0113 | 0.0001 | 0.0000 |
| 5 | 199535814 | 0.4662 | 0.0824 | 0.0363 | 0.0006 | 0.0003 |
| 5 | 208735254 | 0.2567 | 0.0808 | 0.0446 | 0.0008 | 0.0004 |
| 5 | 208735297 | 0.2899 | 0.1249 | 0.0049 | 0.0000 | 0.0000 |
| 5 | 208735527 | 0.2556 | 0.0897 | 0.0308 | 0.0004 | 0.0002 |
| 6 | 33725832 | 0.1031 | 0.1063 | 0.0118 | 0.0001 | 0.0000 |
| 6 | 47640056 | 0.2893 | 0.1124 | 0.0088 | 0.0000 | 0.0000 |
| 6 | 52204826 | 0.4642 | 0.1363 | 0.0026 | 0.0000 | 0.0000 |
| 6 | 53282573 | 0.4424 | 0.0930 | 0.0259 | 0.0003 | 0.0001 |
| 6 | 58482994 | 0.1606 | 0.1486 | 0.0017 | 0.0000 | 0.0000 |
| 6 | 85021804 | 0.1438 | 0.0896 | 0.0259 | 0.0003 | 0.0001 |
| 6 | 85022231 | 0.1410 | 0.0927 | 0.0232 | 0.0002 | 0.0001 |
| 6 | 107114900 | 0.1792 | 0.1107 | 0.0088 | 0.0000 | 0.0000 |
| 6 | 108214241 | 0.2224 | 0.0842 | 0.0391 | 0.0007 | 0.0003 |
| 6 | 142647724 | 0.2828 | 0.0935 | 0.0259 | 0.0003 | 0.0001 |
| 6 | 171939942 | 0.2018 | 0.0956 | 0.0227 | 0.0002 | 0.0001 |
| 7 | 9814174 | 0.4326 | 0.0834 | 0.0346 | 0.0005 | 0.0003 |
| 7 | 14104815 | 0.1068 | 0.0867 | 0.0324 | 0.0004 | 0.0002 |
| 7 | 34979462 | 0.4915 | 0.0783 | 0.0435 | 0.0008 | 0.0004 |
| 7 | 64118834 | 0.2526 | 0.0868 | 0.0318 | 0.0004 | 0.0002 |
| 7 | 64118872 | 0.2537 | 0.0889 | 0.0284 | 0.0003 | 0.0002 |
| 7 | 125647794 | 0.1513 | 0.1193 | 0.0059 | 0.0000 | 0.0000 |
| 7 | 125647809 | 0.1783 | 0.1183 | 0.0062 | 0.0000 | 0.0000 |
| 7 | 125647811 | 0.1825 | 0.1205 | 0.0056 | 0.0000 | 0.0000 |
| 7 | 125647835 | 0.1161 | 0.0781 | 0.0463 | 0.0009 | 0.0005 |
| 7 | 125647852 | 0.1521 | 0.1216 | 0.0055 | 0.0000 | 0.0000 |
| 7 | 153977798 | 0.1508 | 0.0919 | 0.0284 | 0.0003 | 0.0002 |
| 7 | 153977820 | 0.1685 | 0.0921 | 0.0283 | 0.0003 | 0.0002 |
| 7 | 155175818 | 0.4864 | 0.0776 | 0.0472 | 0.0010 | 0.0005 |
| 7 | 155175824 | 0.4873 | 0.0811 | 0.0416 | 0.0007 | 0.0004 |
| 7 | 155175832 | 0.4864 | 0.0776 | 0.0472 | 0.0010 | 0.0005 |
| 7 | 155175835 | 0.4864 | 0.0776 | 0.0472 | 0.0010 | 0.0005 |
| 8 | 8756999 | 0.1667 | 0.0835 | 0.0346 | 0.0005 | 0.0003 |
| 8 | 8757000 | 0.1667 | 0.0835 | 0.0346 | 0.0005 | 0.0003 |
| 8 | 153644935 | 0.2536 | 0.0867 | 0.0346 | 0.0006 | 0.0003 |
| 8 | 153644999 | 0.2492 | 0.0825 | 0.0446 | 0.0008 | 0.0004 |
| 8 | 155611220 | 0.4920 | 0.1558 | 0.0016 | 0.0000 | 0.0000 |
| 8 | 167252034 | 0.4658 | 0.0910 | 0.0318 | 0.0004 | 0.0002 |
| 8 | 173021965 | 0.4991 | 0.0963 | 0.0227 | 0.0002 | 0.0001 |
| 8 | 178016124 | 0.1064 | 0.0791 | 0.0446 | 0.0008 | 0.0004 |
| 9 | 9338142 | 0.2908 | 0.0799 | 0.0450 | 0.0009 | 0.0004 |
| 9 | 9338205 | 0.4071 | 0.0794 | 0.0461 | 0.0009 | 0.0005 |
| 9 | 9338537 | 0.2970 | 0.1004 | 0.0163 | 0.0001 | 0.0001 |
| 9 | 20182088 | 0.1941 | 0.0862 | 0.0326 | 0.0005 | 0.0002 |
| 9 | 65931380 | 0.2679 | 0.1207 | 0.0056 | 0.0000 | 0.0000 |
| 9 | 65931394 | 0.2611 | 0.1148 | 0.0078 | 0.0000 | 0.0000 |
| 9 | 114880830 | 0.1204 | 0.0848 | 0.0326 | 0.0005 | 0.0002 |
| 9 | 130779288 | 0.3088 | 0.0807 | 0.0446 | 0.0008 | 0.0004 |
| 10 | 1786586 | 0.4939 | 0.0870 | 0.0346 | 0.0005 | 0.0003 |
| 10 | 2771013 | 0.1273 | 0.0769 | 0.0461 | 0.0009 | 0.0005 |
| 10 | 65035061 | 0.4694 | 0.1351 | 0.0026 | 0.0000 | 0.0000 |
| 10 | 75581743 | 0.1534 | 0.1121 | 0.0097 | 0.0001 | 0.0000 |
| 10 | 83289114 | 0.4149 | 0.0856 | 0.0335 | 0.0005 | 0.0002 |
| 10 | 85905154 | 0.1388 | 0.0812 | 0.0446 | 0.0008 | 0.0004 |
| 10 | 132099291 | 0.1333 | 0.0866 | 0.0318 | 0.0004 | 0.0002 |
| 10 | 135907217 | 0.2076 | 0.1743 | 0.0005 | 0.0000 | 0.0000 |
| 10 | 135907219 | 0.2377 | 0.1593 | 0.0011 | 0.0000 | 0.0000 |
| 10 | 147739959 | 0.4875 | 0.4700 | 0.0000 | 0.0000 | 0.0000 |
| 10 | 147740010 | 0.2449 | 0.1006 | 0.0188 | 0.0002 | 0.0001 |
| 10 | 147740015 | 0.2463 | 0.1045 | 0.0152 | 0.0001 | 0.0001 |

# Supplementary Information 15. Loci displaying evidence of selection between ex situ and in situ samples identified with BayeScan.

| **Races and municipalities** | **Chr** | **Position (Mb)** | **Log10(PO)** | **q-value** | **alpha** |
| --- | --- | --- | --- | --- | --- |
|  |  |  |  |  |  |
| **All races – All municipalities  (13 pairs)** | 5 | 42792594 | 1.2367 | 0.0314 | 1.7899 |
|  | 6 | 58482994 | 1.0241 | 0.0452 | 1.8704 |
|  | 10 | 135907217 | 1.3870 | 0.0197 | 1.8473 |
|  | 10 | 147739959 | 1000.0000 | 0.0000 | 2.7091 |
| ***Ancho* - All municipalities  (8 pairs)** | 4 | 245085903 | 1.2643 | 0.0258 | 1.9789 |
|  | 6 | 49181017 | 0.6223 | 0.0767 | 1.4943 |
|  | 6 | 52204826 | 1.1753 | 0.0381 | 1.7628 |
|  | 10 | 147739959 | 1000.0000 | 0.0000 | 2.8682 |
| ***Ancho* - Tepoztlán (1 pair)** | 3 | 224165059 | 2.0417 | 0.0090 | 2.2077 |
| ***Ancho* - Totolapan (6 pairs)** | 6 | 49181017 | 2.6980 | 0.0007 | 1.9662 |
|  | 6 | 50784802 | 1.2218 | 0.0125 | 1.7389 |
|  | 6 | 52204826 | 1000.0000 | 0.0000 | 2.1906 |
|  | 6 | 53282573 | 2.3961 | 0.0015 | 1.9752 |
|  | 10 | 147739959 | 1000.0000 | 0.0000 | 3.0560 |

# Supplementary Information 16. Protein coding genes within +/-1 Mb to significant markers identified in DAPC and selection analyses.

| Analysis | Chrom | Physical position (bp) | Gene model | Position (bp) | Gene name |
| --- | --- | --- | --- | --- | --- |
| DAPC - Set | 1 | 239 461 026 | Zm00001d032831 | 239608352 - 239613054 | uce7 - ubiquitin conjugating enzyme7 |
|  |  |  | Zm00001d032832 | 239627717 - 239640745 | arid1 - ARID-transcription factor 1 |
|  |  |  | Zm00001d032850 | 240234642 - 240239267 | pht2 - phosphate transporter protein2 |
|  |  |  | Zm00001d032858 | 240463618 - 240472800 | ks1 - kaurene synthase1 |
| DAPC - Set | 3 | 193 858 128 | Zm00001d043239 | 192793525 - 192802590 | atg18f - autophagy18f |
|  |  |  | Zm00001d043291 | 194717939 - 194725973 | pap1 - purple acid phosphatase1 |
|  |  |  | Zm00001d043293 | 194805524 - 194812236 | cko4 - cytokinin oxidase 4 |
|  |  |  | Zm00001d043258 | 193662256 - 193665537 | IDP2348 |
|  |  |  | Zm00001d043263 | 193694680 - 193704240 | dgk7 - diacylglycerol kinase7 |
|  |  |  | Zm00001d043243 | 192961485 - 192966666 | mrpi2 - MRP interacting2 |
|  |  |  | Zm00001d043244 | 192999229 - 193004686 | aas6 - auxin amido synthetase6 |
| OutFLANK /  BayeScan – *Ancho* Tepoztlán | 3 | 224 165 059 | Zm00001d044259 | 223144810 - 223153404 | hsftf3 - HSF-transcription factor 3 |
|  |  |  | Zm00001d044260 | 223155002 - 223171331 | c3h2 - C3H-transcription factor 32 |
|  |  |  | Zm00001d044272 | 223518403 - 223526903 | bhlh94 - bHLH-transcription factor 94 |
|  |  |  | Zm00001d044278 | 223868324 - 223875657 | rad51c - recombination protein51 gene c |
|  |  |  | Zm00001d044285 | 224211352 - 224218226 | cbl10 - calcineurin B-like10 |
|  |  |  | Zm00001d044301 | 224582450 - 224591060 | prh13 - protein phosphatase homolog13 |
|  |  |  | Zm00001d044311 | 224967972 - 224972256 | mybr110 - MYB-related-transcription factor 110 |
|  |  |  | Zm00001d044315 | 225035151 - 225053176 | wrky7 - WRKY-transcription factor 7 |
| OutFLANK /  BayeScan *Ancho* | 4 | 245 085 903 | Zm00001d053975 | 244504667 - 244519766 | bzr5 - BZR-transcription factor 5 |
|  |  |  | Zm00001d053981 | 244566559 - 244573026 | smk2 - small kernel2 |
|  |  |  | Zm00001d053988 | 244690017 - 244693645 | bzip84 - bZIP-transcription factor 84 |
|  |  |  | Zm00001d053989 | 244753947 - 244766934 | phd5 - PHD-transcription factor 5 |
|  |  |  | Zm00001d053997 | 244820258 - 244831313 | mpk17 - MAP kinase17 |
|  |  |  | Zm00001d053998 | 244828512 - 244835477 | wee1 - wee1 |
|  |  |  | Zm00001d054038 | 245453705 - 245459568 | bhlh128 - bHLH-transcription factor 128 |
|  |  |  | Zm00001d054043 | 245467883 - 245474721 | bip2 - Binding protein homolog2 |
|  |  |  | Zm00001d054044 | 245522181 - 245527259 | cat3 - catalase3 |
| DAPC - Set | 4 | 170 748 561 to  185 951 426 | Zm00001d051859 | 172520975 - 172524674 | cle24 - clavata3/esr-related24 |
|  |  |  | Zm00001d051879 | 173235282 - 173242710 | nactf26 - NAC-transcription factor 26 |
|  |  |  | Zm00001d051891 | 173817077 - 173821160 | lbd24 - LBD-transcription factor 24 |
|  |  |  | Zm00001d051898 | 173924346 - 173929849 | prh1 - ser/thr protein phosphatase1 |
|  |  |  | Zm00001d051945 | 174986019 - 174993537 | phos2 - phosphate transporter2 |
|  |  |  | Zm00001d051976 | 175713376 - 175732030 | ss5 - starch synthase5 |
|  |  |  | Zm00001d051956 | 175256367 - 175261577 | nactf51 - NAC-transcription factor 51 |
|  |  |  | Zm00001d051959 | 175345920 - 175359699 | jmj6 - JUMONJI-transcription factor 6 |
|  |  |  | Zm00001d051961 | 175381901 - 175398833 | jmj9 - JUMONJI-transcription factor 9 |
|  |  |  | Zm00001d051964 | 175483095 - 175497183 | jmj2 - JUMONJI-transcription factor 2 |
|  |  |  | Zm00001d051965 | 175515744 - 175529900 | jmj4 - JUMONJI-transcription factor 4 |
|  |  |  | Zm00001d051981 | 175839487 - 175847670 | gata4 - C2C2-GATA-transcription factor 4 |
|  |  |  | Zm00001d051995 | 176046587 - 176051333 | pcna2 - proliferating cell nuclear antigen2 |
|  |  |  | Zm00001d052010 | 176410962 - 176416389 | umc19 - |
|  |  |  | Zm00001d052018 | 176595615 - 176602043 | dbptf1 - DBP-transcription factor 1 |
|  |  |  | Zm00001d052022 | 176743713 - 176750186 | mha7 - membrane H(+)-ATPase7 |
|  |  |  | Zm00001d052026 | 176837653 - 176841684 | ereb15 - AP2-EREBP-transcription factor 15 |
|  |  |  | Zm00001d052038 | 177038242 - 177044938 | bhlh12 - bHLH-transcription factor 12 |
|  |  |  | Zm00001d052060 | 178017571 - 178024737 | trps10 - trehalose-6-phosphate synthase10 |
|  |  |  | Zm00001d052067 | 178303440 - 178309782 | stk3 - serine-threonine kinase3 |
|  |  |  | Zm00001d052069 | 178363085 - 178368289 | myb16 - MYB-transcription factor 16 |
|  |  |  | Zm00001d052079 | 178763055 - 178777299 | lkrsdh1 - lysine-ketoglutarate reductase/saccharopine dehydrogenase1 |
|  |  |  | Zm00001d052081 | 178934733 - 178940651 | mterf14 - mTERF protein domain21 |
|  |  |  | Zm00001d052087 | 179068681 - 179074322 | ereb14 - AP2-EREBP-transcription factor 14 |
|  |  |  | zma-MIR164f | 179534008 - 179537115 | mir164f - microRNA164f |
|  |  |  | Zm00001d052102 | 179563058 - 179566762 | ereb57 - AP2-EREBP-transcription factor 57 |
|  |  |  | Zm00001d052110 | 179898572 - 179928442 | o1 - opaque endosperm1 |
|  |  |  | Zm00001d052133 | 180557235 - 180564766 | hb83 - Homeobox-transcription factor 83 |
|  |  |  | Zm00001d052136 | 180603177 - 180608529 | acco20 - 1-aminocyclopropane-1-carboxylate oxidase20 |
|  |  |  | Zm00001d052138 | 180621531 - 180631614 | upl14 - ubiquitin-protein ligase14 |
|  |  |  | Zm00001d052139 | 180631025 - 180637340 | nnr5 - nitrate reductase5 |
|  |  |  | Zm00001d052152 | 181351941 - 181355729 | ereb13 - AP2-EREBP-transcription factor 13 |
|  |  |  | Zm00001d052165 | 181551230 - 181557839 | nii2 - nitrite reductase2 |
|  |  |  | Zm00001d052167 | 181558936 - 181562628 | ereb7 - AP2-EREBP-transcription factor 7 |
|  |  |  | Zm00001d052174 | 181714302 - 181719031 | cki6 - cyclin-dependent kinase inhibitor6 |
|  |  |  | Zm00001d052180 | 181855808 - 181865542 | tu1 - tunicate1 |
|  |  |  | Zm00001d052185 | 182577995 - 182585132 | cyc13 - cyclin13 |
|  |  |  | Zm00001d052192 | 182750296 - 182755478 | bhlh36 - bHLH-transcription factor 36 |
|  |  |  | Zm00001d052194 | 182771109 - 182775286 | hsp22 - heat shock protein22 |
|  |  |  | Zm00001d052197 | 183012606 - 183035689 | dek15 - defective kernel15 |
|  |  |  | Zm00001d052200 | 183160557 - 183166840 | fbl41 - F-box protein41 |
|  |  |  | Zm00001d052215 | 183478273 - 183483448 | aprl3 - adenosine 5'-phosphosulfate reductase-like3 |
|  |  |  | Zm00001d052216 | 183513585 - 183518081 | rpl29 - ribosomal protein L29 |
|  |  |  | Zm00001d052220 | 183585560 - 183589159 | vq23 - VQ motif-transcription factor23 |
|  |  |  | Zm00001d052227 | 183818385 - 183824400 | trpp4 - trehalose-6-phosphate phosphatase4 |
|  |  |  | Zm00001d052229 | 183994249 - 183998199 | ereb17 - AP2-EREBP-transcription factor 17 |
|  |  |  | Zm00001d052233 | 184158765 - 184164689 | AY109534 - |
|  |  |  | Zm00001d052240 | 184367541 - 184373294 | ppr5 - pentatricopeptide repeat 5 |
|  |  |  | Zm00001d052243 | 184462669 - 184469079 | lac3 - laccase3 |
|  |  |  | Zm00001d052254 | 184818900 - 184823161 | bhlh155 - bHLH-transcription factor 155 |
|  |  |  | Zm00001d052263 | 185227334 - 185235952 | ss7 - starch synthase7 |
|  |  |  | Zm00001d052269 | 185370137 - 185376598 | PIN-FORMED (PIN proteins) |
|  |  |  | Zm00001d052273 | 185524024 - 185530758 | rop8 - Rho-related protein from plants 8 |
| OutFLANK /  BayeScan - Set | 5 | 42 792 594 | Zm00001d014364 | 42930296 - 42935691 | myb150 - MYB-transcription factor 150 |
|  |  |  | Zm00001d014377 | 43564576 - 43574237 | arftf18 - ARF-transcription factor 18 |
| OutFLANK /  BayeScan *Ancho* BayeScan - *Ancho* Totolapan | 6 | 52 204 826 to  53 282 573 | Zm00001d035835 | 54255739 - 54259728 | ereb72 - AP2-EREBP-transcription factor 72 |
| OutFLANK /  BayeScan - Set | 6 | 58 482 994 | Zm00001d035873 | 58017928 - 58021876 | gras18 - GRAS-transcription factor 18 |
|  |  |  | Zm00001d035903 | 59079985 - 59083659 | abi37 - ABI3-VP1-transcription factor 37 |
|  |  |  | Zm00001d035907 | 59430925 - 59436937 | hb38 - Homeobox-transcription factor 38 |
| DAPC - Set | 7 | 64 118 834 to  64 118 872 | No hits |  |  |
| DAPC - Set | 9 | 109 898 232 | Zm00001d046898 | 109486950 - 109492932 | dps1 - dihydrodipicolinate synthase1 |
|  |  |  | Zm00001d046906 | 109929089 - 109935602 | sbp4 - SBP-domain protein4 |
|  |  |  | Zm00001d046909 | 110046219 - 110060286 | hpt1 - homogentisate phytyltransferase1 |
| OutFLANK /  BayeScan - Set | 10 | 135 907 217 | Zm00001d025989 | 135485993 - 135493526 | iaa43 - Aux/IAA-transcription factor 43 |
|  |  |  | Zm00001d026005 | 135847750 - 135851964 | abi2 - ABI3-VP1-transcription factor 2 |
|  |  |  | Zm00001d026017 | 136071704 - 136077224 | mybr111 - MYB-related-transcription factor 111 |
|  |  |  | Zm00001d026018 | 136173372 - 136181253 | cdpk4 - calcium dependent protein kinase4 |
| OutFLANK /  BayeScan - Set BayeScan - *Ancho* BayeScan - *Ancho* Totolapan | 10 | 147 739 959 | Zm00001d026491 | 147050384 - 147057013 | sbp28 - SBP-transcription factor 28 |
|  |  |  | Zm00001d026498 | 147194518 - 147197808 | clavata3/esr-related19B |
|  |  |  | Zm00001d026501 | 147239129 - 147250881 | gln1 - glutamine synthetase1 |
|  |  |  | Zm00001d026510 | 147363099 - 147366725 | ibh2 - increased leaf inclination1-binding bhlh 2 |
|  |  |  | Zm00001d026515 | 147454886 - 147462191 | vp10 - viviparous10 |
|  |  |  | Zm00001d026518 | 147473169 - 147478475 | bsdtf10 - BSD-transcription factor 10 |
|  |  |  | Zm00001d026537 | 147854036 - 147858373 | hb122 - Homeobox-transcription factor 122 |
|  |  |  | Zm00001d026540 | 147917636 - 147927055 | arftf29 - ARF-transcription factor 29 |
|  |  |  | Zm00001d026542 | 147940912 - 147946998 | glk52 - G2-like-transcription factor 52 |
|  |  |  | Zm00001d026543 | 147948065 - 147959414 | c3h52 - C3H-transcription factor 352 |
|  |  |  | Zm00001d026563 | 148153479 - 148157015 | ereb40 - AP2-EREBP-transcription factor 40 |
|  |  |  | Zm00001d026577 | 148259991 - 148266797 | ccp6 - cysteine protease6 |
|  |  |  | Zm00001d026587 | 148405295 - 148412690 | c3h14 - C3H-transcription factor 314 |

# Supplementary Information 17. QTL coinciding with markers identified with selection analyses.

| Race | Chr | Physical position (bp) | QTL |
| --- | --- | --- | --- |
| *Ancho* – Tepoztlán | 3 | 224 165 059 | ear weight per ear, kernel weight per ear (Yi *et al.*, 2019) |
| *Ancho* | 4 | 245 085 903 | - |
| All (set) | 5 | 42 792 594 | ear diameter (Sabadin *et al.*, 2008)  grain yield (Alves Lima *et al.*, 2006)  hundred-kernel weight, kernel number per row (Chen *et al*., 2016) kernel width (Liu *et al.*, 2014; Hui et al., 2015; Chen *et al.*, 2016; Raihan *et al.*, 2016) metaQTL for ear related traits, kernel related traits and grain yield (Chen *et al.*, 2017) metaQTL for grain weight (Pan *et al*., 2017; Zhou *et al*., 2020) |
| *Ancho*  *Ancho* - Totolapan | 6 | 52 204 826 | grain yield per plant (Su *et al.*, 2017) kernel width (Chen *et al*., 2016) metaQTL for ear related traits, kernel related traits and grain yield (Chen *et al*., 2017)  metaQTL for ear weight per plant, grain weight and grain yield (Zhou *et al.*, 2020) |
| *Ancho* - Totolapan | 6 | 53 282 573 |  |
| All (set) | 6 | 58 482 994 | kernel width (Zhang *et al*., 2014) metaQTL for ear related traits, kernel related traits and grain yield (Chen *et al*., 2017)  metaQTL for ear weight per plant, grain weight and grain yield (Zhou *et al.*, 2020) |
| All (set) | 10 | 135 907 217 | kernel width (Hui *et al*., 2015) metaQTL for ear related traits, kernel related traits and grain yield (Chen *et al*., 2017) |
| All (set)  *Ancho*  *Ancho* - Totolapan | 10 | 147 739 959 | ear length, kernel number per row (Huo *et al*., 2016)  cob length, kernel length (Liu *et al*., 2019)  kernel volume, kernel weight (Zhang *et al*., 2014)  kernel width (Hui et al., 2015; Zhu *et al*., 2018)  metaQTL for anthesis-silking interval, kernel weight, ear length and grain yield (Zhao *et al.*, 2018) metaQTL for ear related traits, kernel related traits and grain yield (Chen *et al*., 2017) |

# References

Alves Lima M de L, Lopes de Souza C, Vieira Bento DA, Pereira de Souza A, Carlini-Garcia LA (2006). Mapping QTL for Grain Yield and Plant Traits in a Tropical Maize Population. *Mol Breed* **17**: 227–239.

Chen L, An Y, Li Y-X, Li C, Shi Y, Song Y, *et al.* (2017). Candidate Loci for Yield-Related Traits in Maize Revealed by a Combination of MetaQTL Analysis and Regional Association Mapping. *Front Plant Sci* **8**: 2190.

Chen J, Zhang L, Liu S, Li Z, Huang R, Li Y, *et al.* (2016). The Genetic Basis of Natural Variation in Kernel Size and Related Traits Using a Four-Way Cross Population in Maize. *PLoS One* **11**: e0153428.

Hartl DL, Clark AG (1997). *Principles of Population Genetics* (Sinauer, Ed.). Sunderland, MA.

Hui G, Wen G, Liu X, Yang H, Luo Q, Song H, *et al.* (2015). Quantitative trait locus analysis for kernel width using maize recombinant inbred lines. *Genet Mol Res* **14**: 14496–14502.

Huo D, Ning Q, Shen X, Liu L, Zhang Z (2016). QTL Mapping of Kernel Number-Related Traits and Validation of One Major QTL for Ear Length in Maize (A Zhang, Ed.). *PLoS One* **11**: e0155506.

Liu Y, Wang L, Sun C, Zhang Z, Zheng Y, Qiu F (2014). Genetic analysis and major QTL detection for maize kernel size and weight in multi-environments. *Theor Appl Genet* **127**: 1019–1037.

Liu Y, Yi Q, Hou X, Hu Y, Li Y, Yu G, *et al.* (2019). Identification of quantitative trait loci for kernel-related traits and the heterosis for these traits in maize (Zea mays L.). *Mol Genet Genomics* **295**: 121–133.

Pan L, Yin Z, Huang Y, Chen J, Zhu L, Zhao Y, *et al.* (2017). QTL for maize grain yield identified by QTL mapping in six environments and consensus loci for grain weight detected by meta-analysis (T Lübberstedt, Ed.). *Plant Breed* **136**: 820–833.

Pixley K V, Salinas-Garcia GE, Hall A, Kropff M, Ortiz C, Bouvet LC, *et al.* (2018). CIMMYT’s Seeds of Discovery Initiative: Harnessing Biodiversity for Food Security and Sustainable Development. *Indian J Plant Genet Resour* **31**: 1.

Raihan MS, Liu J, Huang J, Guo H, Pan Q, Yan J (2016). Multi-environment QTL analysis of grain morphology traits and fine mapping of a kernel-width QTL in Zheng58 × SK maize population. *Theor Appl Genet* **129**: 1465–1477.

Sabadin PK, Lopes De Souza Júnior C, Pereira De Souza A, Garcia AAF (2008). QTL mapping for yield components in a tropical maize population using microsatellite markers. *Hereditas* **145**: 194–203.

Su C, Wang W, Gong S, Zuo J, Li S, Xu S (2017). High Density Linkage Map Construction and Mapping of Yield Trait QTLs in Maize (Zea mays) Using the Genotyping-by-Sequencing (GBS) Technology. *Front Plant Sci* **8**: 706.

Yi Q, Liu Y, Hou X, Zhang X, Li H, Zhang J, *et al.* (2019). Genetic dissection of yield-related traits and mid-parent heterosis for those traits in maize (Zea mays L.). *BMC Plant Biol* **19**: 1–20.

Zhang Z, Liu Z, Hu Y, Li W, Fu Z, Ding D, *et al.* (2014). QTL analysis of kernel-related traits in maize using an immortalized F 2 population (JC Nelson, Ed.). *PLoS One* **9**: e89645.

Zhao X, Peng Y, Zhang J, Fang P, Wu B (2018). Identification of QTLs and meta-QTLs for seven agronomic traits in multiple maize populations under well-watered and water-stressed conditions. *Crop Sci* **58**: 507–520.

Zhou Z, Li G, Tan S, Li D, Weiß TM, Wang X, *et al.* (2020). A QTL atlas for grain yield and its component traits in maize (Zea mays). *Plant Breed* **139**: 562–574.

Zhu X-M, Shao X-Y, Pei Y-H, Guo X-M, Li J, Song X-Y, *et al.* (2018). Genetic Diversity and Genome-Wide Association Study of Major Ear Quantitative Traits Using High-Density SNPs in Maize. *Front Plant Sci* **9**: 966.
